# Psychological Outcomes following a nurse-led Preventative Psychological Intervention for critically ill patients (POPPI): protocol for a cluster-randomised clinical trial of a complex intervention

**DOI:** 10.1136/bmjopen-2017-020908

**Published:** 2018-02-08

**Authors:** Alvin Richards-Belle, Paul R Mouncey, Dorothy Wade, Chris R Brewin, Lydia M Emerson, Richard Grieve, David A Harrison, Sheila Harvey, David Howell, Monty Mythen, Zia Sadique, Deborah Smyth, John Weinman, John Welch, Kathryn M Rowan

**Affiliations:** 1 Clinical Trials Unit, Intensive Care National Audit & Research Centre (ICNARC), London, UK; 2 Critical Care Department, University College London Hospitals NHS Foundation Trust, London, UK; 3 Research Department of Clinical, Educational & Health Psychology, University College London, London, UK; 4 Centre for Experimental Medicine, Queen’s University Belfast, Belfast, UK; 5 Department of Health Services Research and Policy, London School of Hygiene & Tropical Medicine, London, UK; 6 NIHR Biomedical Research Centre, University College London/University College London Hospitals, Institute of Sport Exercise and Health (ISEH), London, UK; 7 Institute of Pharmaceutical Science, King’s College London, London, UK

**Keywords:** Critical care, Intensive care, ICU, mental health, PTSD, psychological intervention

## Abstract

**Introduction:**

Acute psychological stress, as well as unusual experiences including hallucinations and delusions, are common in critical care unit patients and have been linked to post-critical care psychological morbidity such as post-traumatic stress disorder (PTSD), depression and anxiety. Little high-quality research has been conducted to evaluate psychological interventions that could alleviate longer-term psychological morbidity in the critical care unit setting. Our research team developed and piloted a nurse-led psychological intervention, aimed at reducing patient-reported PTSD symptom severity and other adverse psychological outcomes at 6 months, for evaluation in the POPPI trial.

**Methods and analysis:**

This is a multicentre, parallel group, cluster-randomised clinical trial with a staggered roll-out of the intervention. The trial is being carried out at 24 (12 intervention, 12 control) NHS adult, general, critical care units in the UK and is evaluating the clinical effectiveness and cost-effectiveness of a nurse-led preventative psychological intervention in reducing patient-reported PTSD symptom severity and other psychological morbidity at 6 months. All sites deliver usual care for 5 months (baseline period). Intervention group sites are then trained to carry out the POPPI intervention, and transition to delivering the intervention for the rest of the recruitment period. Control group sites deliver usual care for the duration of the recruitment period. The trial also includes a process evaluation conducted independently of the trial team.

**Ethics and dissemination:**

This protocol was reviewed and approved by the National Research Ethics Service South Central - Oxford B Research Ethics Committee (reference: 15/SC/0287). The first patient was recruited in September 2015 and results will be disseminated in 2018. The results will be presented at national and international conferences and published in peer reviewed medical journals.

**Trial registration number:**

ISRCTN53448131; Pre-results.

**Strengths and limitations of this study**

- POPPI is the first, large, multicentre cluster-randomised clinical trial evaluating a complex intervention commencing in the critical care unit and aimed at reducing longer-term psychological morbidity for critical care survivors in the UK.
- POPPI has strong patient and public involvement, with former critical care patients involved in the development of the research question and intervention, training of key trial staff and as members of oversight committees.
- The trial has an embedded economic evaluation and an independent process evaluation.
- The primary outcome is patient-reported and it is anticipated that there may be 20%–25% missing data.

## Introduction

More than 170 000 patients are admitted to adult, general, critical care units in the National Health Service (NHS) each year.^1^ It has been estimated that approximately 50% of critically ill patients suffer serious emotional distress, and up to two-thirds have unusual experiences such as hallucinations and delusions, while in the unit.^2 3^ Emotional distress, including severe symptoms of anxiety, low mood and panic, may be caused by a range of stressful experiences that are common in the critical care unit: fear of dying; invasive treatments such as mechanical ventilation; pain and discomfort; inability to communicate; and terrifying hallucinatory delusions.^2 4–6^ The hallucinations and delusions of critical care unit patients have been linked to delirium, the provision and withdrawal of sedative and other psychoactive drugs, effects of illness (such as sepsis), immobility, and sensory and sleep deprivation.^3 5 7^ Critical care unit hallucinations frequently have horrifying themes such as conspiracy to kill by staff, torture, poisoning, demons, extortion or organ theft;^8^ thus a vicious cycle of stress, confusion and terror is common for critical care unit patients.

Experiencing acute psychological stress in the critical care unit, or having frequent memories of hallucinations and delusions, are among the identified risk factors for longer-term post-critical care post-traumatic stress disorder (PTSD), depression, anxiety or cognitive impairment.^5 9–13^ Systematic reviews of survivors of critical care identified high rates of PTSD (median 20%)^4 14^ and depression (median 30%),^15 16^ months or years after leaving critical care. Patients who develop serious long-term psychological distress are at much higher risk of further physical morbidities and mortality,^17–19^ representing a serious burden to patients, to their carers and to NHS.^20 21^

Little high-quality research has been conducted to evaluate psychological interventions that could alleviate the emotional distress experienced by patients in critical care, with a view to preventing longer-term psychological morbidity.^22^ The introduction of valid psychological assessment tools for use with critical care patients (eg, the intensive care psychological assessment tool (IPAT))^23^ has made evaluation of psychological interventions more feasible. Research informing the best timing to provide psychological interventions suggests that postdischarge (eg, at 6 weeks^24^ or at outpatient follow-up clinics^21^) may be too late, and earlier intervention could be more beneficial.^25^ In today’s NHS, practitioner psychologists are still a scarce resource, and a more pragmatic approach would be to standardise brief evidence-based psychological interventions to be carried out by existing critical care staff, who would be given the necessary training.

Our research team developed and piloted a nurse-led psychological intervention for critical care unit patients which commences within the unit and is based on up-to-date evidence concerning psychological techniques that are effective in: (A) reducing acute emotional distress, (B) reducing the impact of unusual experiences such as hallucinations and delusions and (C) preventing PTSD after a trauma. These are all psychological problems commonly associated with admission to critical care. We hypothesised that these existing evidence-based psychological interventions could be modified to reduce the stress and trauma experienced by critical care unit patients, and be delivered by specially trained, well-motivated critical care nurses. There is an urgent need to evaluate their effectiveness in the critical care unit setting.

This protocol was informed by the Psychological Outcomes following a nurse-led Preventative Psychological Intervention for critically ill patients (POPPI) feasibility study (ISRCTN61088114) which looked at feasibility and acceptability of both the intervention and the trial procedures.

## Methods

### Aim and objectives

The POPPI trial aims to evaluate the clinical effectiveness and cost-effectiveness of a complex nurse-led preventative psychological intervention in reducing patient-reported PTSD symptom severity, and other reported psychological problems, at 6 months. The specific objectives are:

- to evaluate the effect of the complex intervention on patient-reported PTSD symptom severity and other psychological outcomes and quality of life at 6 months; and
- to estimate, in an integrated economic analysis, the cost-effectiveness of the intervention.

In addition, an integrated process evaluation will be conducted to assess the fidelity, dose and reach of the implementation of the intervention, and to identify important contextual factors to better understand how the intervention may work.

### Study design and setting

The POPPI trial is a multicentre, parallel group, cluster-randomised clinical trial (cluster-RCT), conducted in 24 NHS adult, general, critical care units.

### Intervention

The POPPI trial will evaluate a intervention comprising three elements:

1. Creating a therapeutic environment in critical care
2. Three stress support sessions for patients screened as acutely stressed
3. Relaxation and recovery programme for patients screened as acutely stressed

An education package (two training courses and associated materials) to train critical care staff to carry out the three elements has been developed and piloted by our research team and will be described in detail elsewhere (paper under review for publication).

### Sites

NHS adult, general, critical care units (‘sites’) are eligible to participate if they are able to commit to the following criteria:

- show that recruitment, data collection and delivery of the intervention are feasible;
- adherence to cluster-randomisation;
- Identify two joint-Principal Investigators (PIs) (a nurse and a doctor) to lead the trial locally;
- agree, where possible, to recruit all eligible patients and to maintain a screening and enrolment log; and
- continue active participation in the Case Mix Programme (CMP)—the national clinical audit for adult critical care in England, Wales and Northern Ireland coordinated by the Intensive Care National Audit & Research Centre (ICNARC).

Sites who piloted the intervention during the POPPI feasibility study are not eligible to participate in the trial.

#### Randomisation

The 24 sites will be randomly assigned to either the intervention group (n=12) or the control group (n=12), by the ICNARC CTU, using a restricted randomisation approach to ensure balance across the groups in terms of geographical location, hospital teaching status and size of unit. For each group of eight sites, the individual sites will be randomised (4:4) in their second month of recruitment. It is necessary to randomise on a cluster (‘site’), rather than individual level to avoid contamination of usual care, as it would not be possible to restrict parts of the intervention to individual patients.

Sites randomised to the intervention group are responsible for selecting three POPPI nurses based on a person specification which includes:

- Registered nurse with ≥3 years critical care clinical experience
- Effective communicator, with patients and their families, colleagues and collaborators
- Able to work flexibly
- Interested in improving psychological care of patients
- Organised and able to manage a busy schedule

#### Timeline

**Figure 1:**
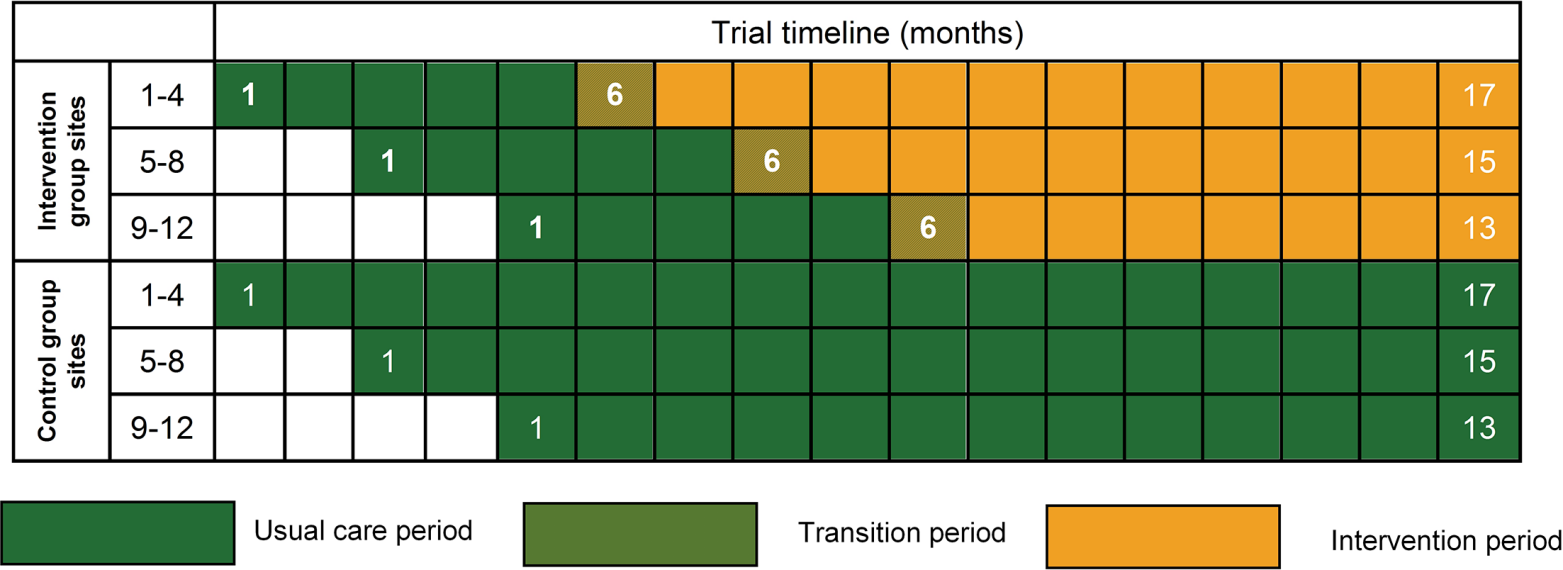
POPPI cluster-randomised clinical trial schedule.

The 24 sites will open to recruitment in three groups of eight sites at 2-month intervals and recruit patients over a 17-month period (see figure 1). Control group sites will deliver usual care for the duration of the recruitment period. Intervention group sites will deliver usual care from months 1 to 5. Usual care is defined as patients receiving psychological support or treatment at the discretion of the treating clinician(s) following standard practice at their site.

**Figure 2:**
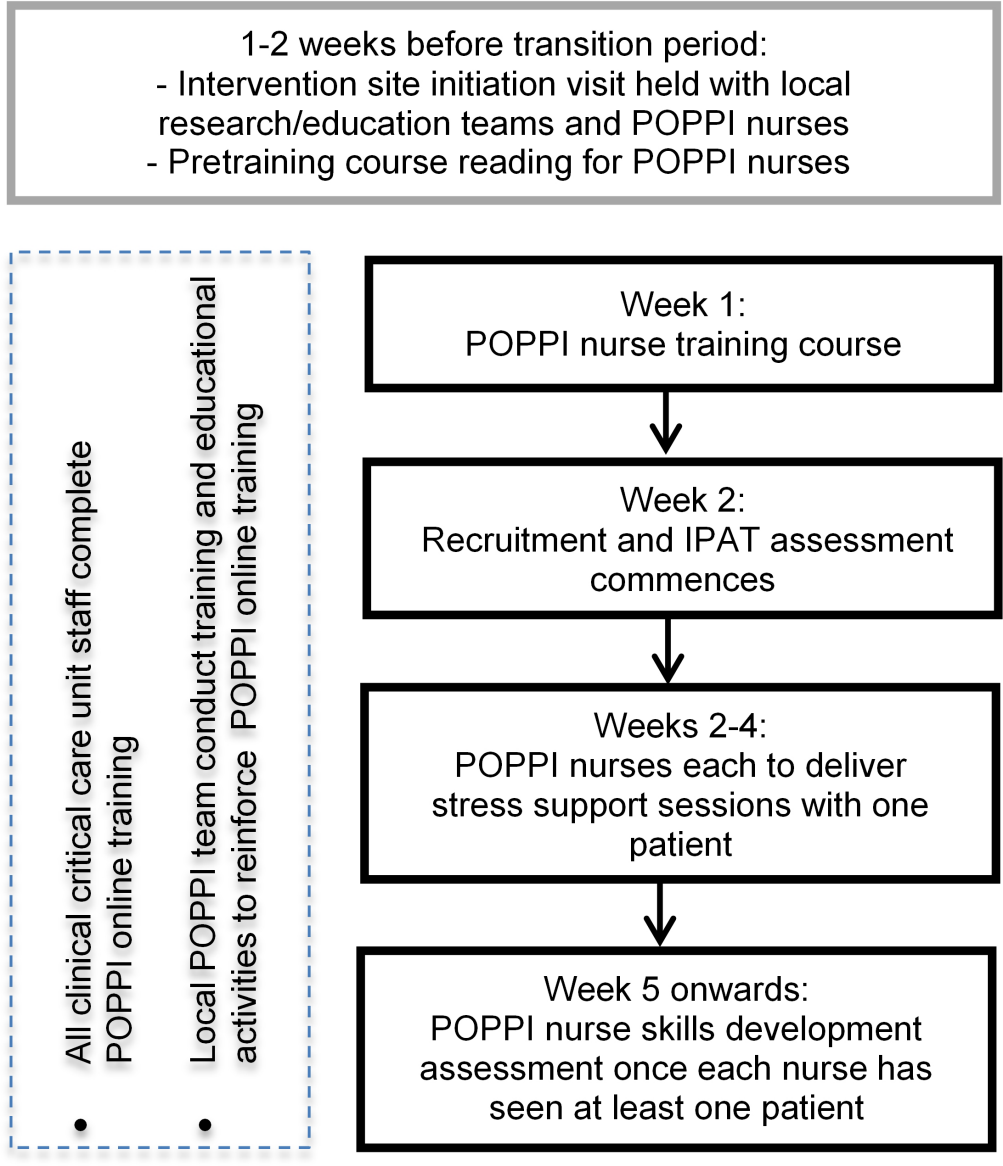
Site timeline during transition period. IPAT, intensive care psychological assessment tool.

After month 5, intervention group sites will undergo a 1-month transition period, during which they will transition from delivering usual care to delivering the intervention (see figure 2). At the beginning of the transition period, all POPPI nurses at a site will attend a 3-day POPPI nurse training course. Following the training course and completion of a local intervention site initiation visit, the POPPI nurses and local education/research teams will commence delivery of the POPPI intervention. During the transition period, each POPPI nurse should deliver stress support sessions to at least one recruited patient, identified (using the IPAT) as being stressed and at high risk of psychological morbidity. In parallel, the POPPI nurses and local education/research teams will encourage culture change in their unit to create a therapeutic environment. This will be done by ensuring all clinical critical care staff complete the POPPI online training and through other educational activities (eg, seminars and short presentations, bedside teaching and display of materials reinforcing key messages from the POPPI online training). At the end of this transition period, the POPPI nurses will undergo a skills development assessment. Following the transition period, the intervention will be delivered until the end of the recruitment period.

#### POPPI nurse training course

The POPPI nurse training course is a 3-day central course to train POPPI nurses in their new role. The focus of the course is on learning and practising the skills required to deliver the stress support sessions with patients. The course was designed by the trial team in consultation with experts in medical education and cognitive behavioural therapy training, and is delivered by a psychologist, two senior nurses and a research assistant.

The course will cover:

- Understanding critical care patients’ stress (including patient representative talks and videos)
- Learning the skills needed to deliver stress support sessions
- Content of each of the stress support sessions
- Observing (in person and expert videos) example stress support sessions
- Practising each of the stress support sessions
- Using the patient booklet to create personal action plans
- Debriefing and support arrangements

Associated materials include: a precourse theory booklet, a training folder and POPPI nurse training manual on the three stress support sessions; a tablet computer with a ‘relax and recover’ app for patients to use with help from nurses and family; and a self-help booklet and digital video disc (DVD) for patients to take home.

The POPPI nurse role also includes; encouraging clinical staff in their units to complete the POPPI online training; promoting the screening of patients with IPAT; and teaching good communication skills and psychological care (reinforcing key messages from the POPPI online training) at the bedside. These tasks will be completed in conjunction with the research/education teams at each intervention site as a team approach and training will be provided by the trial team at intervention site initiation visits held locally.

#### Debriefing and support for POPPI nurses

All POPPI nurses will be allocated a trainer from the POPPI training team to provide debriefing and support following the training course. Debriefing and support will focus on enhancing nurses’ skills and discussing patients’ cases. The first debriefing and support session will be carried out once a POPPI nurse has delivered stress support sessions to their first patient. Once all POPPI nurses at the site have delivered stress support sessions to at least one patient each, the POPPI training team will visit the POPPI nurses in their units to offer further support. During the visit, POPPI nurses will also undergo a skills development assessment, to ensure they meet the required standards for delivering the sessions. If necessary, further support and training will be offered prior to the delivery of further sessions with patients. POPPI nurses will continue to receive debriefing and support either via telephone call or site visit.

#### Creating a therapeutic environment

Each intervention group site will create a therapeutic environment by encouraging culture change in their unit. This will be facilitated by ensuring all clinical critical care unit staff complete the POPPI online training and by teaching and modelling good communication skills and psychological care at the bedside. The POPPI online training is an online training course designed to aid the creation of a calm, less stressful environment by reducing stressors in the environment and using good communication with patients. The POPPI online training takes approximately 30 min to complete and comprises five sections (understanding the stresses of intensive care patients, reducing stress and fear in patients, communicating with distressed patients, inspiring patients with confidence and hope, and summary and assessment). Local research teams will enumerate all clinical critical care staff at the start of the transition period, and then monthly thereafter to ensure new staff members are registered for the POPPI online training.

In addition, intervention group sites will ensure that POPPI materials are clearly displayed (eg, posters) and distributed (eg, pocket cards) throughout the unit.

### Patients

Patients admitted to participating units will be routinely screened against the eligibility criteria:

#### Inclusion criteria

- Age 18 years or greater
- Greater than 48 hours in the critical care unit
- Receipt of level 3 critical care (for any period of time) during first 48 hours in the critical care unit
- Between +1 and −1 on the Richmond Agitation Sedation Scale^26^
- Glasgow Coma Scale Score of 15
- English-speaking
- Ability to communicate orally

#### Exclusion criteria

- Pre-existing chronic cognitive impairment, such as dementia
- Pre-existing psychotic illness, such as schizophrenia
- Pre-existing chronic PTSD
- Receiving end-of-life care
- Previously recruited to POPPI

**Figure 3:**
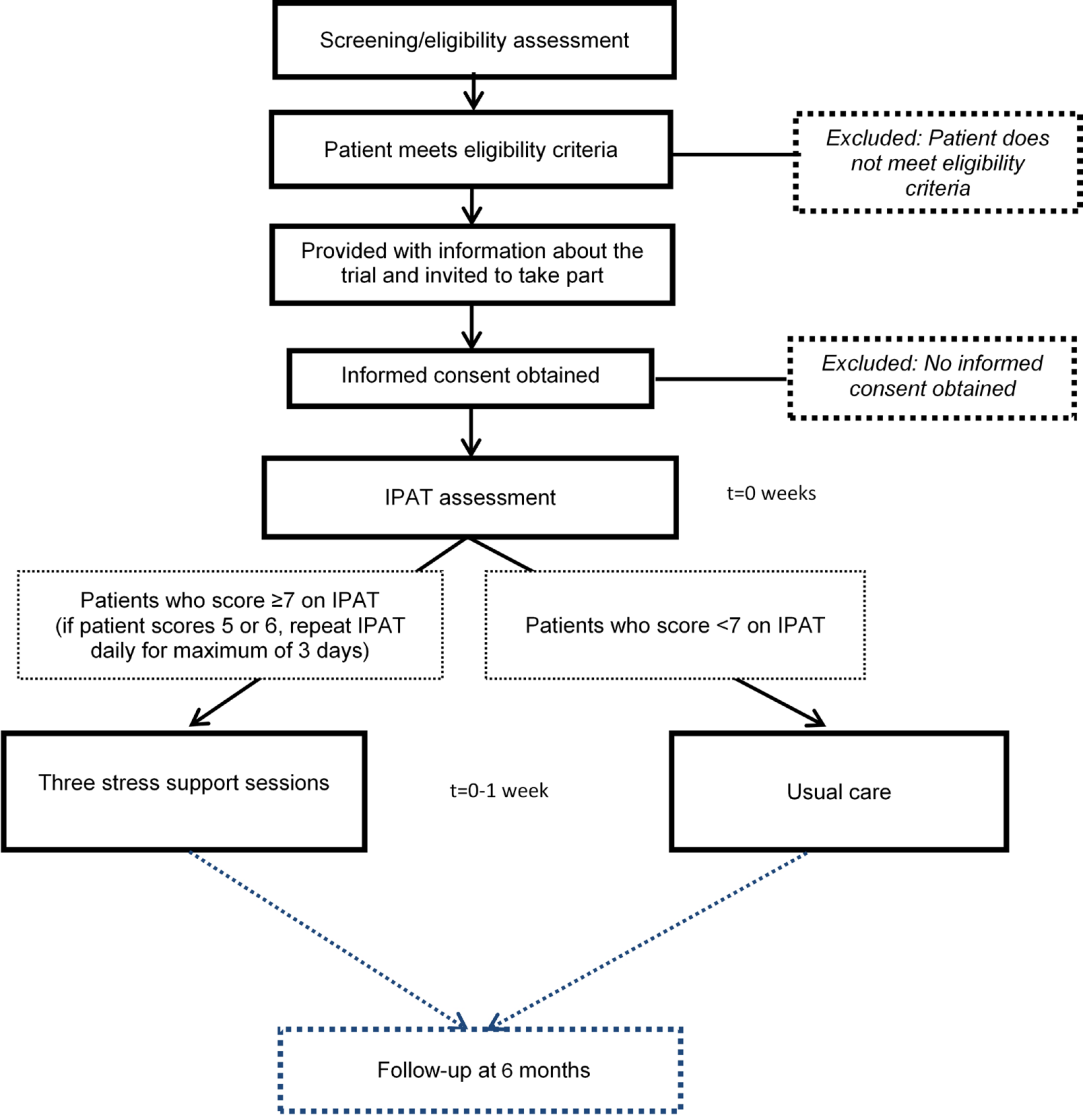
Patient timeline during the intervention period. IPAT, intensive care psychological assessment tool.

Patients who meet the eligibility criteria in the unit will be approached and provided with written and verbal information about POPPI by a member of the local research team. Potential participants will be given the opportunity to ask questions and time to discuss the trial with family or friends before making their decision. After the person seeking consent is satisfied that the information has been understood and questions have been answered, they will invite the potential participant to sign the consent form. In providing informed consent, participants are agreeing for the trial team to have access to their medical records for data collection and to receive a follow-up questionnaire at 6 months. In addition, participants recruited at intervention group sites from month 6 onwards (figure 1) will be offered the option to provide consent to receive an assessment with IPAT and subsequent stress support sessions (if applicable). Figure 3 shows the timeline for a patient recruited at an intervention group site from month 6 onwards.

On entry into the study, the participant’s general practitioner (GP) will be informed, by letter, of their recruitment into POPPI.

#### IPAT assessment

IPAT is a validated screening tool used to detect acute psychological stress and unusual experiences such as hallucinations in critically ill patients.^23^ Consenting, eligible patients at intervention group sites will be assessed using IPAT by a trained staff member as soon as possible, but within 48 hours of consent being provided. A patient is deemed highly stressed if they score seven or more on IPAT and should be referred, as soon as possible, to a POPPI nurse to receive the three stress support sessions. Patients who score less than 7 on IPAT will continue to receive usual care as determined by the treating clinician(s). If the patient scores 5 or 6 on IPAT they will be reassessed daily, for a maximum of 3 days, until they either leave the critical care unit or the score drops below 5.

#### Stress support sessions

The main objectives of the stress support sessions are for nurses to develop a trusting relationship with patients, so patients can discuss concerns which they might feel embarrassed or worried about communicating, and to reduce emotional distress. There are three common components to each stress support session: starting the session; building rapport; and finishing the session. In addition, each session is structured as follows:

- Stress support session 1—‘helping patients understand and cope with stress’
  - Normalise reactions
  - Encourage communication
  - Teach coping strategies
- Stress support session 2—‘managing frightening thoughts from critical care’
  - Stress reactions
  - Explain stressful thinking
  - Teach ‘check out my fear’ technique
- Stress support session 3—‘creating confidence and hope for a good recovery’
  - Summarise and review
  - Action plan
  - Future expectations

The three stress support sessions are to be delivered by the same POPPI nurse ideally within 1 week, with the first stress support session starting as soon as possible, but within 48 hours following IPAT assessment. Each session lasts approximately 30 min and ideally (at least) the first session is delivered in the critical care unit, but sessions can be delivered elsewhere in the hospital if the patient moves. If a patient shows signs of distress or fatigue during the session, then the session can be stopped and a new visit can be arranged at a more appropriate time. The State-Trait Anxiety Inventory (STAI)^27^ will be used to assess a patient’s anxiety prior to session one (at baseline) and at the end of stress support session 3. If a patient is showing serious signs of distress at the end of their three sessions, their medical team will be informed.

#### Follow-up

All participants will be sent a follow-up questionnaire by the ICNARC CTU 6 months postrecruitment. The questionnaire contains the PTSD Symptom Scale – Self-Report version (PSS-SR),^28^ the Hospital Anxiety and Depression Scale (HADS)^29^ the EuroQoL health questionnaire (EQ-5D-5L)^30^ and a health services and resource use questionnaire. Questionnaire packs include a self-addressed stamped envelope and a pen for ease of return. Non-responders will be telephoned 3 weeks later to check whether they have received the questionnaire and, if preferable, they will be given the option to complete the questionnaire over the telephone. If completed questionnaires received at the ICNARC CTU indicate the presence of signs of serious stress, anxiety or depression, a referral letter from DW (lead clinical investigator) will be sent to the patient’s GP or the local PIs at the recruiting site.

### Outcomes

#### Primary outcomes

The primary outcome for the clinical evaluation is patient-reported PTSD symptom severity at 6 months, measured using PSS-SR,^28^ which conforms to the Diagnostic and Statistical Manual of Mental Disorders (fourth edition) diagnostic criteria for PTSD and which has been validated for use in critical care unit survivors.

The primary outcomes for the economic evaluation will be incremental costs, quality-adjusted life years (QALYs) and net monetary benefit at 6 months.

#### Secondary outcomes

Secondary outcomes are:

- days alive and free from sedation to day 30;
- duration of critical care unit stay;
- PSS-SR greater than 18 points at 6 months;^31^
- anxiety and depression at 6 months, measured using HADS;^29^
- health-related quality of life (HRQoL) at 6 months, measured by EQ-5D-5L^30^ and
- estimated lifetime cost-effectiveness.

### Data collection

Table 1 shows the patient data collection schedule. The following data are collected by local research teams for all patients while in hospital:

- Patient details (identifiers, sociodemographics)
- Clinical/baseline data (date/time of critical care unit admission and consent, eligibility criteria, quality of life score, STAI^27^ score, prior delirium (assessed by the Confusion Assessment Method for the Intensive Care Unit (CAM-ICU),^32^ documented pre-existing anxiety or depression)
- Critical care unit stay data (duration of: delirium (assessed by CAM-ICU), sedatives, anxiolytics, anaesthetics, sleep medications, antipsychotics, analgesics, antidepressants, vasoactive agents and mechanical ventilation)
- Hospital discharge data (discharge status, date of discharge/death)
- POPPI intervention data (for patients recruited at intervention group sites during the intervention period—IPAT Scores, delivery of stress support sessions, STAI Score after session 3)

**Table 1:** Patient data collection schedule

	Baseline (at point of recruitment)	End of critical care unit stay	Intervention group sites—during transition and intervention periods			Six months postrecruitment
			Before SSS-I	During sessions	After SSS-III	
Collected inhospital						
Patient details	✓					
Clinical/baseline data	✓					
Critical care unit stay		✓				
IPAT assessment			✓			
SSS delivery				✓		
STAI	✓				✓	
Collected via follow-up questionnaires sent to patients						
PSS-SR						✓
HADS						✓
EQ-5D-5L						✓
Health service and resource use						✓

EQ-5D-5L, EuroQoL Health questionnaire; HADS, Hospital Anxiety and Depression Scale; IPAT, Intensive care Psychological Assessment Tool; PSS-SR, Post-traumatic Stress Disorder Symptom Scale - Self Report version; SSS, stress support session(s); STAI, State-Trait Anxiety Inventory.

Follow-up data (PSS-SR, HADS, EQ-5D-5L, health services and resource use) are collected via the patient follow-up questionnaire at 6 months postrecruitment. In addition, data will be linked to CMP and will include demographics, surgical status, acute severity of illness and duration of organ support, and duration of critical care unit stay. Support for the collection and use of patient-identifiable data has been approved for CMP by the Patient Information Advisory Group (PIAG) under Section 251 of the NHS Act 2006—approval number: PIAG 2-10(f)/2005. Survival at 6 months will be ascertained through NHS Digital. All data are managed in accordance with ICNARC CTU standard operating procedures.

The process evaluation will consider both quantitative and qualitative data. Mixed-methods data will be collected for all three component parts of the intervention, to elucidate the degree to which the intervention was delivered as intended. Quantitative data will include the rate of online training uptake, treatment fidelity of the stress support sessions, and routinely collected screening and recruitment data. At intervention group sites, qualitative data will be collected in the form of researcher observations, interviews with staff and structured field notes. An independent researcher will observe and discuss the delivery of the intervention with the POPPI nurses and wider critical care unit staff, exploring clinician experiences including those relating to barriers and facilitators to the delivery of the intervention. The process evaluation will also incorporate visits to a purposively selected sample of control group sites. Qualitative data will be collected to understand wider trial processes including strategies to ensure/promote recruitment, and any changes in unit practice from baseline.

### Analysis

An overview of the planned analyses for the POPPI trial is provided below. The full statistical analysis plan will be submitted for publication ahead of database lock.

#### Clinical evaluation

The primary analysis for the clinical evaluation will determine if there is a significant difference in the mean PSS-SR at 6 months between patients recruited during the intervention period at intervention sites compared with patients recruited at control sites using a generalised linear mixed model (GLMM) at the individual patient level (patients nested within sites and time periods) including a random effect of site and a fixed effect of period (baseline or intervention), and adjusted for site-level factors included within the restricted randomisation algorithm.

For the primary outcome, the link function will be the identity link (ie, linear regression) and standard errors will be estimated using a jackknife variance estimate, which has been demonstrated in simulation studies to maintain the size of the test.^33^

A secondary analysis will adjust for prespecified baseline factors associated with poor psychological outcome (eg, sedation) and ability to resource and deliver the intervention (eg, size of critical care unit, teaching status) at both patient and site levels. Results of GLMMs will be reported as differences in means, 95% CIs and P values.

Analyses of secondary outcomes will be conducted using GLMMs, with the identity link (ie, linear regression) for continuous secondary outcomes, reported as differences in means, and the logit link (ie, logistic regression) for binary secondary outcomes, reported as ORs.

The above analyses will evaluate the effectiveness of the intervention among all patients meeting the inclusion criteria and consenting to follow-up, based on the intention-to-treat principle. A further secondary analysis will use structural mean models with an instrumental variable of allocated treatment to estimate the efficacy (adherence-adjusted causal effect) of the stress support sessions among those patients consenting to psychological assessment and stress support sessions, assessed as being at high risk of psychological morbidity and receiving stress support sessions.^34^

#### Economic evaluation

A cost-effectiveness analysis (CEA) will be undertaken to assess the relative cost-effectiveness of the intervention versus usual care. Resource use and outcome data will be used to report cost-effectiveness at 6 months and to project the lifetime cost-effectiveness of each strategy.

The cost analysis will take a health and personal health services perspective. Resource use data will be combined with unit costs from the NHS Payment by Results database and from local Trust Finance Departments, to report the total costs per patient at 6 months for intervention versus usual care.^35 36^

HRQoL data from the EQ-5D-5L questionnaires at 6 months will be combined with survival data using linear interpolation to report QALYs at 6 months. CEA will follow the intention-to-treat principle and report the mean (95% CI) incremental costs, QALYs and net monetary benefit at 6 months.

CEA will use multilevel linear regression models that allow for clustering^37^ including a random effect of site and a fixed effect of period. The analysis will adjust for prespecified baseline covariates at both patient level and site level.

Lifetime cost-effectiveness will be projected by encapsulating the relative effects of the alternative strategies on long-term survival and HRQoL, combining extrapolations from the patient survival data, with external evidence on long-term survival and HRQoL.^38 39^ The long-term survival of patients will be extrapolated from the cluster-RCT data by fitting alternative parametrical survival curves (eg, Weibull, exponential, lognormal, log logistic and Gompertz) to the observed survival data. The method of parametrical extrapolation of survival for the base case will be chosen on the basis of model fit and plausibility when compared with age-gender matched general population survival.^40^ Survival will then be extrapolated according to the chosen parametrical function for the duration of years that parametrical curves predict excess mortality compared with the age-gender matched general population, after which we will assume that all-cause death rates were those of the age-gender matched general population. In the base case, quality of life calculated at 6 months will be assumed to apply to each subsequent year of life, after allowing for decrements in quality of life according to advancing age. We will project lifetime costs by applying morbidity costs estimated at 6 months over the period of excess mortality. Predicted survival and HRQoL will be combined to report lifetime QALYs, and to project lifetime incremental costs, incremental QALYs and incremental net benefits for the alternative strategies of care. Sensitivity analyses will test whether the results are robust to methodological assumptions (eg, specification of the statistical model, extrapolation approach, alternative HRQoL assumptions and learning curve effects).

#### Process evaluation

The process evaluation data will be analysed using a combination of qualitative and quantitative methods to measure and understand the reason for any variation in the delivery of the intervention across sites. Interview data will be transcribed and analysed using a seven-stepped framework approach^41^ which includes coding the data, developing and applying an analytical framework, and producing data summaries. A sample of transcripts will be double-coded and reviewed by another independent member of the research team to ensure trustworthiness and confirmability. Data summaries will be interpreted and used to construct overall explanations of the data by two members of the research team.

Analysis of the process evaluation data will be conducted before the outcome evaluation to avoid any bias in the interpretation of the data, and to generate hypotheses that may be subsequently tested in statistical analyses of integrated process and outcome data. The process evaluation data will be combined with the trial outcome data to uncover the relationship between the variation in intervention delivery and trial outcomes.

### Power calculation

#### Pretrial power calculation

The power calculation was completed using the approach of Hussey and Hughes (2007)^33^ to achieve 90% power to detect a reduction from 6 points to 3.1 points (P<0.05) in the mean PSS-SR at 6 months, and was based on the following assumptions:

- Mean (6) and SD (7.5) of the PSS-SR were taken from patients in the feasibility study
- Estimated intracluster correlation (ICC) of 0.138—between-site coefficient of variation 0.5 corresponding to between-site SD of 3 (conservative estimate as no multicentre data available).^42^ Note: the inclusion of a baseline recruitment period means that the sample size calculation is less sensitive to the degree of clustering^33^
- Treatment effect of a reduction of 2.9 points on PSS-SR based on: reliable change index for the PSS-SR of 8.6 points^43^ being observed in 40% of eligible patients in the intervention periods assessed as being at high risk of psychological morbidity using IPAT, with 16% of recruiting patients declining the intervention
- Harmonic mean of the number of patients completing follow-up (52 per site per annum—corresponding to 22 in a 5-month period) based on data from CMP

With the design and the above assumptions, the estimated total number of patients recruited (based on CMP data) for the RCT would be 1914 patients from the 24 sites. It is anticipated that 438 will be assessed using IPAT, of which 175 (40%) will be assessed as being at high risk of psychological morbidity and receive the stress support sessions.

#### Final review of assumptions in pretrial power calculation

During recruitment, in consultation with the Trial Steering Committee (TSC) and Data Monitoring and Ethics Committee (DMEC), a review of assumptions underlying the pretrial power calculation was conducted once outcome data were available for patients recruited during the 5-month baseline period in both the intervention group and control group sites. This review, undertaken using data available on 9 August 2016, identified the following re-estimations of the assumptions:

- Mean (10.3) and SD (10.8) of PSS-SR
- ICC of 0.087 (95% CI 0 to 0.192) (with mean, SD and ICC estimated using all available data from a previous observational study, the feasibility study and the baseline period of the cluster-RCT)
- Treatment effect of a reduction of 4.2 points on PSS-SR—estimated by retaining the same effect size as a multiple of the within-site SD
- Harmonic mean of the number of patients completing follow-up (30.7 per site per annum —corresponding to 12.8 in a 5-month period) estimated using observed data from the baseline period

This review established that the planned design had an anticipated 78% power under the observed parameter estimates (allowing for uncertainty in the between-site variation, between 73% and 85% power).

Consequently, the decision was taken to extend recruitment in all sites to the end of planned recruitment for the final group of eight sites (corresponding to an harmonic mean of 16.5 patients completing follow-up per site during the intervention period, allowing for the variation from 5 months to 9 months duration across sites, figure 1). With this extension to recruitment, the planned design had an anticipated 85% power (allowing for uncertainty in the between-site variation, between 79% and 91% power). It was anticipated that, with this extension to recruitment, the estimated total number of patients recruited would be 1378. Recruitment continued to be monitored to ensure 1378 or more patients were recruited. A final decision to extend recruitment by an additional 2 months in all sites was taken to ensure this minimum number was achieved. A protocol amendment was implemented to reflect this review of assumptions and the extension to the recruitment period.

### Oversight

The Trial Management Group (TMG) is responsible for the management of POPPI and is led by KMR (chief investigator) and PRM (senior researcher). In addition, TMG comprises the trial investigators and relevant ICNARC Clinical Trials Unit (CTU) staff, who meet regularly to discuss the progress of the trial. ICNARC is trial sponsor (contact details available on http://www.icnarc.org), and ICNARC CTU is responsible for day-to-day trial management, is the data custodian, and will conduct central and on-site monitoring of sites and data. POPPI is managed according to the principles of the International Conference on Harmonisation Good Clinical Practice. ICNARC CTU will act to preserve patient confidentiality and will not disclose or reproduce any information by which patients could be identified. Any patient identifiable data leaving sites will be encrypted to ensure anonymity. All procedures for handling, processing, storing and destroying data are compliant with the Data Protection Act 1998.

TSC and DMEC have been convened as trial oversight committees. TSC is independently chaired by Professor Sallie Lamb and includes critical care clinicians, psychologists, patient and public involvement representatives, along with the chief investigator and lead clinical investigator. TSC monitors trial progress and at least 75% of members are independent. The independent DMEC is chaired by Professor Marion Campbell and also includes an experienced critical care clinician and an experienced statistician. DMEC is independent of both the trial team, sponsor and TSC, and operates under the DAMOCLES Charter,^44^ reporting to TSC, making recommendations on the continuation, or not, of the trial.

## Trial status

This paper presents the protocol (V.2.2, 6 March 2017) for the first, large, multicentre cluster-RCT evaluating a complex intervention commencing in the critical care unit and aimed at reducing longer-term psychological morbidity for critical care survivors in the UK. The full p rotocol (including amendments) is available on the NIHR website. 45 The first participant was recruited in September 2015. At the time of first manuscript submission, data collection for the trial was ongoing and due to be complete in December 2017. The trial results will be disseminated in 2018 through presentations at national and international conferences and publication in peer reviewed medical journals.

## Supplementary Material

Reviewer comments

Author's manuscript
