# Supplementary material for: Psychological Outcomes following a nurse-led Preventative Psychological Intervention for critically ill patients (POPPI): protocol for a cluster-randomised clinical trial of a complex intervention
Source: BMJ Open. 2018 Feb 8;8(2):e020908. doi: 10.1136/bmjopen-2017-020908 (PMC5879925; doi:10.1136/bmjopen-2017-020908)
Supplement: Author's manuscript [file bmjopen-2017-020908.draft_revisions.pdf]

BMJ Open is committed to open peer review. As part of this commitment we make the peer review history of every article we publish publicly available.

When an article is published we post the peer reviewers' comments and the authors' responses online. We also post the versions of the paper that were used during peer review. These are the versions that the peer review comments apply to.

The versions of the paper that follow are the versions that were submitted during the peer review process. They are not the versions of record or the final published versions. They should not be cited or distributed as the published version of this manuscript.

BMJ Open is an open access journal and the full, final, typeset and author-corrected version of record of the manuscript is available on our site with no access controls, subscription charges or pay-per-view fees (http://bmjopen.bmj.com).

If you have any questions on BMJ Open's open peer review process please email info.bmjopen@bmj.com

BMJ Open

Psychological Outcomes following a nurse-led Preventative Psychological Intervention for critically ill patients (POPPI) – Protocol for a cluster randomised clinical trial of a complex intervention

Journal:	*BMJ Open*
Manuscript ID	bmjopen-2017-020908
Article Type:	Protocol
Date Submitted by the Author:	30-Nov-2017
Complete List of Authors:	Richards-Belle, Alvin; Intensive Care National Audit & Research Centre, Clinical Trials Unit<br>Mouncey, Paul; Intensive Care National Audit & Research Centre, Clinical Trials Unit<br>Wade, Dorothy; UCLH NHS Foundation Trust, Critical Care<br>Brewin, Chris; University College London, Research Department of Clinical, Educational & Health Psychology<br>Emerson, Lydia; Queen's University Belfast, Centre for Experimental Medicine<br>Grieve, Richard; London School of Hygiene & Tropical Medicine, Department of Health Services Research and Policy<br>Harrison, David; Intensive Care National Audit & Research Centre, Clinical Trials Unit<br>Harvey, Sheila; Intensive Care National Audit & Research Centre, Clinical Trials Unit<br>Howell, David; University College London Hospitals NHS Foundation Trust, Critical Care Department<br>Mythen, Monty; University College London/ University College London Hospitals NIHR Biomedical Research Centre, Institute of Sport Exercise and Health (ISEH)<br>Sadique, Zia; London School of Hygiene and Tropical Medicine, Department of Health Services Research and Policy<br>Smyth, Deborah; University College London Hospitals NHS Foundation Trust, Critical Care Department<br>Weinman, John; Kings College London, Institute of Pharmaceutical Sciences & Institute of Psychiatry<br>Welch, John; University College London Hospitals NHS Foundation Trust, Critical Care Department<br>Rowan, Kathryn; Intensive Care National Audit & Research Centre, Clinical Trials Unit
Keywords:	INTENSIVE & CRITICAL CARE, Adult intensive & critical care < INTENSIVE & CRITICAL CARE, MENTAL HEALTH, PTSD, Protocol

For peer review only

TITLE

Psychological Outcomes following a nurse-led Preventative Psychological Intervention for critically ill patients (POPPI) – Protocol for a cluster randomised clinical trial of a complex intervention

AUTHORS

Alvin Richards-Belle, Trial Manager[1]

Paul R Mouncey, Senior Researcher[1]

Dorothy Wade, Health Psychologist[2]

Chris R Brewin, Professor of Clinical Psychology[3]

Lydia M Emerson, MRC-HTMR Research Fellow[4]

Richard Grieve, Professor of Health Economics Methodology[5]

David A Harrison, Head Statistician[1]

Sheila Harvey, Senior Research Fellow[1]

David Howell, Divisional Clinical Director[2]

Monty Mythen, Professor of Anaesthesia and Critical Care[6]

Zia Sadique, Assistant Professor in Health Economics[5]

Deborah Smyth, Senior Research Nurse[2]

John Weinman, Professor of Health Psychology as Applied to Medicine[7]

John Welch, Consultant Nurse, Critical Care and Critical Care Outreach[2]

Kathryn M Rowan, Director of Scientific and Strategic Development/Clinical Trials Unit Director[1]

On behalf of the POPPI Trial Investigators

[1]Clinical Trials Unit, Intensive Care National Audit & Research Centre (ICNARC), Napier House, 24 High Holborn, London, WC1V 6AZ

[2]Critical Care Department, University College London Hospitals NHS Foundation Trust, 235 Euston Road, London, NW1 2BU

[3]Research Department of Clinical, Educational & Health Psychology, University College London, Gower Street, London, WC1E 6BT

[4]Centre for Experimental Medicine, Queen's University Belfast, 97 Lisburn Road, Belfast, BT9 7BL

[5]London School of Hygiene & Tropical Medicine, 15-17 Tavistock Place, London, WC1H 9SH

[6]University College London/ University College London Hospitals NIHR Biomedical Research Centre c/o Institute of Sport Exercise and Health (ISEH) 170 Tottenham Court Road, London, W1T 7HA

[7]Institute of Pharmaceutical Science, King's College London, Franklin-Wilkins Building, London, SE1 9NH

For peer review only

Corresponding author:

Professor Kathryn Rowan

Email address: Kathy.Rowan@icnarc.org

Telephone: 020 7269 9277

Fax: 020 7831 6879

Keywords:

Critical care, Intensive care, ICU, mental health, PTSD, psychological intervention

Word count: Abstract (282), Main manuscript (5,501)

For peer review only

ABSTRACT

Introduction

Acute psychological stress, as well as unusual experiences including hallucinations and delusions, are common in critical care unit patients and have been linked to post-critical care psychological morbidity such as post-traumatic stress disorder (PTSD), depression and anxiety. Little high-quality research has been conducted to evaluate psychological interventions that could alleviate longer-term psychological morbidity in the critical care unit setting. Our research team developed and piloted a nurse-led psychological intervention, aimed at reducing patient-reported PTSD symptom severity and other adverse psychological outcomes at six months, for evaluation in the POPPI trial.

Methods and analysis

This is a multi-centre, parallel group, cluster-randomised clinical trial with a staggered roll out of the intervention. The trial is being carried out at 24 (12: intervention, 12: control) NHS adult, general, critical care units in the UK and is evaluating the clinical and cost-effectiveness of a nurse-led preventative psychological intervention in reducing patient-reported PTSD symptom severity and other psychological morbidity at six months. All sites deliver usual care for five months (baseline period). Intervention group sites are then trained to carry out the POPPI intervention, and transition to delivering the intervention for the rest of the recruitment period. Control group sites deliver usual care for the duration of the recruitment period. The trial also includes a process evaluation conducted independently of the trial team.

Ethics and dissemination

This Protocol was reviewed and approved by NRES Committee South Central - Oxford B Research Ethics Committee (reference: 15/SC/0287). The first patient was recruited in September 2015 and results will be disseminated in 2018. The results will be presented at national and international conferences and published in peer reviewed medical journals.

Trial registration

ISRCTN53448131 – prospectively registered on 15 July 2015.

Strengths and limitations of this study

- POPPI is the first, large, multi-centre cluster-randomised clinical trial evaluating a complex intervention commencing in the critical care unit and aimed at reducing longer-term psychological morbidity for critical care survivors in the UK.
- POPPI has strong patient and public involvement, with former critical care patients involved in the development of the research question and intervention, training of key trial staff, and as members of oversight committees.
- The trial has an embedded economic evaluation and an independent process evaluation.
- The primary outcome is patient-reported and it is anticipated that there may be 20-25% missing data.

For peer review only - http://bmjopen.bmj.com/site/about/guidelines.xhtml
For peer review only

INTRODUCTION

More than 170,000 patients are admitted to adult, general, critical care units in the National Health Service (NHS) each year.[1] It has been estimated that approximately 50% of critically ill patients suffer serious emotional distress, and up to two-thirds have unusual experiences such as hallucinations and delusions, while in the unit.[2 3] Emotional distress, including severe symptoms of anxiety, low mood and panic, may be caused by a range of stressful experiences that are common in the critical care unit: fear of dying; invasive treatments such as mechanical ventilation; pain and discomfort; inability to communicate; and terrifying hallucinatory delusions.[2 4-6] The hallucinations and delusions of critical care unit patients have been linked to delirium, the provision and withdrawal of sedative and other psychoactive drugs, effects of illness (such as sepsis), immobility, and sensory and sleep deprivation.[3 5 7] Critical care unit hallucinations frequently have horrifying themes such as conspiracy to kill by staff, torture, poisoning, demons, extortion or organ theft[8]; thus a vicious cycle of stress, confusion, and terror is common for critical care unit patients.

Experiencing acute psychological stress in the critical care unit, or having frequent memories of hallucinations and delusions, are among the identified risk factors for longer-term post-critical care post-traumatic stress disorder (PTSD), depression, anxiety or cognitive impairment.[5 9-13] Systematic reviews of survivors of critical care identified high rates of PTSD (median 20%[4 14]) and depression (median 30%[15 16]), months or years after leaving critical care. Patients who develop serious long-term psychological distress are at much higher risk of further physical morbidities and mortality,[17-19] representing a serious burden to patients, to their carers and to the NHS.[20 21]

Little high-quality research has been conducted to evaluate psychological interventions that could alleviate the emotional distress experienced by patients in critical care, with a view to preventing longer-term psychological morbidity.[22] The introduction of valid psychological assessment tools for use with critical care patients (e.g. the Intensive care Psychological Assessment Tool (IPAT)[23]) has made evaluation of psychological interventions more feasible. Research informing the best timing to provide psychological interventions suggests that post-discharge (e.g. at six weeks[24] or at outpatient follow-up clinics[21]) may be too late, and earlier intervention could be more beneficial.[25] In today's NHS, practitioner psychologists are still a scarce resource, and a more pragmatic approach would be to standardise brief evidence-based psychological interventions to be carried out by existing critical care staff, who would be given the necessary training.

Our research team developed and piloted a nurse-led psychological intervention for critical care unit patients which commences within the unit and is based on up-to-date evidence concerning psychological techniques that are effective in: a) reducing acute emotional distress b) reducing the impact of unusual experiences such as hallucinations and delusions and c) preventing PTSD after a trauma. These are all psychological problems commonly associated with admission to critical care. We hypothesised that these existing evidence-based psychological interventions could be modified to reduce the stress and trauma experienced by critical care unit patients, and be delivered by specially trained, well-motivated critical care nurses. There is an urgent need to evaluate their effectiveness in the critical care unit setting.

This protocol was informed by the POPPI feasibility study (ISRCTN61088114) which looked at feasibility and acceptability of both the intervention and the trial procedures.

For peer review only - http://bmjopen.bmj.com/site/about/guidelines.xhtml

For peer review only

METHODS

Aim and objectives

The POPPI trial aims to evaluate the clinical and cost-effectiveness of a complex nurse-led preventative psychological intervention in reducing patient-reported PTSD symptom severity, and other reported psychological problems, at six months. The specific objectives are:

- to evaluate the effect of the complex intervention on patient-reported PTSD symptom severity and other psychological outcomes and quality of life at six months; and
- to estimate, in an integrated economic analysis, the cost-effectiveness of the intervention.

In addition, an integrated process evaluation will be conducted to assess the fidelity, dose and reach of the implementation of the intervention, and to identify important contextual factors to better understand how the intervention may work.

Study design and setting

The POPPI trial is a multi-centre, parallel group, cluster-randomised clinical trial (cluster-RCT), conducted in 24 NHS adult, general, critical care units.

Intervention

The POPPI trial will evaluate a intervention comprising three elements:

1) Creating a therapeutic environment in critical care
2) Three stress support sessions for patients screened as acutely stressed
3) Relaxation and recovery programme for patients screened as acutely stressed

An education package (two training courses and associated materials) to train critical care staff to carry out the three elements has been developed and piloted by our research team and will be described in detail elsewhere (paper under review for publication).

Sites

NHS adult, general, critical care units ('sites') are eligible to participate if they are able to commit to the following criteria:

- show that recruitment, data collection, and delivery of the intervention are feasible;
- adherence to cluster-randomisation;
- Identify two joint-Principal Investigators (a nurse and a doctor) to lead the trial locally;
- agree, where possible, to recruit all eligible patients and to maintain a Screening and Enrolment Log; and
- continue active participation in the Case Mix Programme (CMP) – the national clinical audit for adult critical care in England, Wales and Northern Ireland coordinated by the Intensive Care National Audit & Research Centre (ICNARC).

For peer review only - http://bmjopen.bmj.com/site/about/guidelines.xhtml

For peer review only

Sites who piloted the intervention during the POPPI feasibility study are not eligible to participate in the trial.

Randomisation

The 24 sites will be randomly assigned to either the intervention group (N=12) or the control group (N=12), by the ICNARC CTU, using a restricted randomisation approach to ensure balance across the groups in terms of geographical location, hospital teaching status and size of unit. For each group of eight sites, the individual sites will be randomised (4:4) in their second month of recruitment. It is necessary to randomise on a cluster ('site'), rather than individual, level to avoid contamination of usual care, as it would not be possible to restrict parts of the intervention to individual patients.

Sites randomised to the intervention group are responsible for selecting three POPPI nurses based on a person specification which includes:

- Registered nurse with ≥ three years critical care clinical experience
- Effective communicator, with patients and their families, colleagues and collaborators
- Able to work flexibly
- Interested in improving psychological care of patients
- Organised and able to manage a busy schedule

Timeline

The 24 sites will open to recruitment in three groups of eight sites at two month intervals and recruit patients over a 17-month period (see Figure 1). Control group sites will deliver usual care for the duration of the recruitment period. Intervention group sites will deliver usual care from months one to five. Usual care is defined as patients receiving psychological support or treatment at the discretion of the treating clinician(s) following standard practice at their site.

After month five, intervention group sites will undergo a one month transition period, during which they will transition from delivering usual care to delivering the intervention (see Figure 2). At the beginning of the transition period, all POPPI nurses at a site will attend a three-day POPPI nurse training course. Following the training course and completion of a local intervention site initiation visit, the POPPI nurses and local education/research teams will commence delivery of the POPPI intervention. During the transition period, each POPPI nurse should deliver stress support sessions to at least one recruited patient, identified (using the IPAT) as being stressed and at high risk of psychological morbidity. In parallel, the POPPI nurses and local education/research teams will encourage culture change in their unit to create a therapeutic environment. This will be done by ensuring all clinical critical care staff complete the POPPI online training and through other educational activities (e.g. seminars and short presentations, bedside teaching, and display of materials reinforcing key messages from the POPPI online training). At the end of this transition period, the POPPI nurses will undergo a skills development assessment. Following the transition period, the intervention will be delivered until the end of the recruitment period.

POPPI nurse training course

The POPPI nurse training course is a three-day central course to train POPPI nurses in their new role. The focus of the course is on learning and practising the skills required to deliver the stress support sessions with

For peer review only - http://bmjopen.bmj.com/site/about/guidelines.xhtml

patients. The course was designed by the trial team in consultation with experts in medical education and CBT training, and is delivered by a psychologist, two senior nurses and a research assistant.

The course will cover:

- Understanding critical care patients' stress (including patient representative talks and videos)
- Learning the skills needed to deliver stress support sessions
- Content of each of the stress support sessions
- Observing (in person and expert videos) example stress support sessions
- Practising each of the stress support sessions
- Using the patient booklet to create personal action plans
- Debriefing and support arrangements

Associated materials include: a pre-course theory booklet, a training folder and POPPI nurse training manual on the three stress support sessions; a tablet computer with a "relax and recover" app for patients to use with help from nurses and family; and a self-help booklet and DVD for patients to take home.

The POPPI nurse role also includes; encouraging clinical staff in their units to complete the POPPI online training; promoting the screening of patients with the IPAT; and teaching good communication skills and psychological care (reinforcing key messages from the POPPI online training) at the bedside. These tasks will be completed in conjunction with the research/education teams at each intervention site as a team approach and training will be provided by the trial team at intervention site initiation visits held locally.

Debriefing and support for POPPI nurses

All POPPI nurses will be allocated a trainer from the POPPI training team to provide debriefing and support following the training course. Debriefing and support will focus on enhancing nurses' skills and discussing patients' cases. The first debriefing and support session will be carried out once a POPPI nurse has delivered stress support sessions to their first patient. Once all POPPI nurses at the site have delivered stress support sessions to at least one patient each, the POPPI training team will visit the POPPI nurses in their units to offer further support. During the visit, POPPI nurses will also undergo a skills development assessment, to ensure they meet the required standards for delivering the sessions. If necessary, further support and training will be offered prior to the delivery of further sessions with patients. POPPI nurses will continue to receive debriefing and support either via telephone call or site visit.

Creating a therapeutic environment

Each intervention group site will create a therapeutic environment by encouraging culture change in their unit. This will be facilitated by ensuring all clinical critical care unit staff complete the POPPI online training and by teaching and modelling good communication skills and psychological care at the bedside. The POPPI online training is an online training course designed to aid the creation of a calm, less stressful environment by reducing stressors in the environment and using good communication with patients. The POPPI online training takes approximately 30 minutes to complete and comprises five sections (understanding the stresses of intensive care patients, reducing stress and fear in patients, communicating with distressed patients, inspiring patients with confidence and hope, and summary and assessment). Local research teams will

For peer review only - http://bmjopen.bmj.com/site/about/guidelines.xhtml

enumerate all clinical critical care staff at the start of the transition period, and then monthly thereafter to ensure new staff members are registered for the POPPI online training.

In addition, intervention group sites will ensure that POPPI materials are clearly displayed (e.g. posters) and distributed (e.g. pocket cards) throughout the unit.

Patients

Patients admitted to participating units will be routinely screened against the eligibility criteria:

Inclusion criteria

- Age 18 years or greater
- Greater than 48 hours in the critical care unit
- Receipt of Level 3 critical care (for any period of time) during first 48 hours in the critical care unit
- Between +1 and -1 on the Richmond Agitation Sedation Scale[26]
- Glasgow Coma Scale score of 15
- English-speaking
- Ability to communicate orally

Exclusion criteria

- Pre-existing chronic cognitive impairment, such as dementia
- Pre-existing psychotic illness, such as schizophrenia
- Pre-existing chronic PTSD
- Receiving end-of-life care
- Previously recruited to POPPI

Patients who meet the eligibility criteria in the unit will be approached and provided with written and verbal information about POPPI by a member of the local research team. Potential participants will be given the opportunity to ask questions and time to discuss the trial with family or friends before making their decision. After the person seeking consent is satisfied that the information has been understood and questions have been answered, they will invite the potential participant to sign the Consent Form. In providing informed consent, participants are agreeing for the trial team to have access to their medical records for data collection and to receive a follow-up questionnaire at six months. In addition, participants recruited at intervention group sites from month six onwards (Figure 1) will be offered the option to provide consent to receive an assessment with the IPAT and subsequent stress support sessions (if applicable). Figure 3 shows the timeline for a patient recruited at an intervention group site from month six onwards.

On entry into the study, the participant's general practitioner (GP) will be informed, by letter, of their recruitment into POPPI.

IPAT assessment

The IPAT is a validated screening tool used to detect acute psychological stress and unusual experiences such as hallucinations in critically ill patients.[23] Consenting, eligible patients at intervention group sites will be

For peer review only - http://bmjopen.bmj.com/site/about/guidelines.xhtml

assessed using the IPAT by a trained staff member as soon as possible, but within 48 hours of consent being provided. A patient is deemed highly stressed if they score seven or more on the IPAT and should be referred, as soon as possible, to a POPPI nurse to receive the three stress support sessions. Patients who score less than seven on the IPAT will continue to receive usual care as determined by the treating clinician(s). If the patient scores five or six on the IPAT they will be reassessed daily, for a maximum of three days, until they either leave the critical care unit or the score drops below five.

Stress support sessions

The main objectives of the stress support sessions are for nurses to develop a trusting relationship with patients, so patients can discuss concerns which they might feel embarrassed or worried about communicating, and to reduce emotional distress. There are three common components to each stress support session: Starting the session; Building Rapport; and Finishing the session. In addition, each session is structured as follows:

- Stress support session one – "helping patients understand and cope with stress"
    - Normalise reactions
    - Encourage communication
    - Teach coping strategies

- Stress support session two – "managing frightening thoughts from critical care"
    - Stress reactions
    - Explain stressful thinking
    - Teach "check out my fear" technique

- Stress support session three – "creating confidence and hope for a good recovery"
    - Summarise and review
    - Action plan
    - Future expectations

The three stress support sessions are to be delivered by the same POPPI nurse ideally within one week, with the first stress support session starting as soon as possible, but within 48 hours following IPAT assessment. Each session lasts approximately 30 minutes and ideally (at least) the first session is delivered in the critical care unit, but sessions can be delivered elsewhere in the hospital if the patient moves. If a patient shows signs of distress or fatigue during the session, then the session can be stopped and a new visit can be arranged at a more appropriate time. The State Trait Anxiety Inventory[27] will be used to assess a patient's anxiety prior to session one (at baseline) and at the end of stress support session three. If a patient is showing serious signs of distress at the end of their three sessions, their medical team will be informed.

Follow-up

All participants will be sent a follow-up questionnaire by the ICNARC CTU six months post-recruitment. The questionnaire contains the PTSD Symptom Scale – Self-Report version (PSS-SR)[28], the Hospital Anxiety and

For peer review only - http://bmjopen.bmj.com/site/about/guidelines.xhtml

Depression Scale (HADS)[29] the EuroQoL health questionnaire (EQ-5D-5L)[30] and a health services and resource use questionnaire. Questionnaire packs include a self-addressed stamped envelope and a pen for ease of return. Non-responders will be telephoned three weeks later to check whether they have received the questionnaire and, if preferable, they will be given the option to complete the questionnaire over the telephone. If completed questionnaires received at the ICNARC CTU indicate the presence of signs of serious stress, anxiety or depression, a referral letter from Dr Dorothy Wade (Lead Clinical Investigator) will be sent to the patient's GP or the local PIs at the recruiting site.

Outcomes

Primary outcomes

The primary outcome for the clinical evaluation is patient-reported PTSD symptom severity at six months, measured using the PSS-SR[28], which conforms to the Diagnostic and Statistical Manual of Mental Disorders (fourth edition, DSM-IV) diagnostic criteria for PTSD and which has been validated for use in critical care unit survivors.

The primary outcomes for the economic evaluation will be incremental costs, quality-adjusted life years (QALYs) and net monetary benefit at six months.

Secondary outcomes

Secondary outcomes are:

- days alive and free from sedation to day 30;
- duration of critical care unit stay;
- PSS-SR greater than 18 points at six months[31];
- anxiety and depression at six months, measured using HADS[29];
- health-related quality of life (HRQoL) at six months, measured by the EuroQol (EQ-5D-5L)[30] questionnaire; and
- estimated lifetime cost-effectiveness.

Data collection

Table one shows the patient data collection schedule. The following data are collected by local research teams for all patients whilst in-hospital:

- Patient details (identifiers, socio-demographics)
- Clinical/baseline data (date/time of critical care unit admission and consent, eligibility criteria, quality of life score, State-Trait Anxiety Inventory (STAI)[27] score, prior delirium (assessed by the Confusion Assessment Method for the Intensive Care Unit (CAM-ICU)[32]), documented pre-existing anxiety or depression
- Critical care unit stay data (duration of: delirium (assessed by CAM-ICU), sedatives, anxiolytics, anaesthetics, sleep medications, antipsychotics, analgesics, anti-depressants, vasoactive agents and mechanical ventilation)
- Hospital discharge data (discharge status, date of discharge/death)
- POPPI Intervention data (for patients recruited at intervention group sites during the intervention period - IPAT scores, delivery of stress support sessions, STAI score after session three)

For peer review only - http://bmjopen.bmj.com/site/about/guidelines.xhtml

Follow-up data (PSS-SR, HADS, EQ-5D-5L, health services and resource use) are collected via the patient follow-up questionnaire at six months post-recruitment. In addition, data will be linked to the CMP and will include demographics, surgical status, acute severity of illness and duration of organ support and duration of critical care unit stay. Support for the collection and use of patient identifiable data has been approved for the CMP by the Patient Information Advisory Group (PIAG) under Section 251 of the NHS Act 2006 – Approval Number: PIAG 2-10(f)/2005. Survival at six months will be ascertained through NHS Digital. All data is managed in accordance with ICNARC CTU Standard Operating Procedures.

The process evaluation will consider both quantitative and qualitative data. Mixed-methods data will be collected for all three component parts of the intervention, to elucidate the degree to which the intervention was delivered as intended. Quantitative data will include the rate of online training uptake, treatment fidelity of the stress support sessions, and routinely collected screening and recruitment data. At intervention group sites, qualitative data will be collected in the form of researcher observations, interviews with staff and structured field notes. An independent researcher will observe and discuss the delivery of the intervention with the POPPI nurses and wider critical care unit staff, exploring clinician experiences including those relating to barriers and facilitators to the delivery of the intervention. The process evaluation will also incorporate visits to a purposively selected sample of control group sites. Qualitative data will be collected to understand wider trial processes including strategies to ensure/promote recruitment, and any changes in unit practice from baseline.

For peer review only

ANALYSIS

An overview of the planned analyses for the POPPI trial is provided below. The full Statistical Analysis Plan will be submitted for publication ahead of database lock.

Clinical evaluation

The primary analysis for the clinical evaluation will determine if there is a significant difference in the mean PSS-SR at six months between patients recruited during the intervention period at intervention sites compared with patients recruited at control sites using a generalised linear mixed model (GLMM) at the individual patient level (patients nested within sites and time periods) including a random effect of site and a fixed effect of period (baseline or intervention), and adjusted for site-level factors included within the restricted randomisation algorithm.

For the primary outcome, the link function will be the identity link (i.e. linear regression) and standard errors will be estimated using a jackknife variance estimate, which has been demonstrated in simulation studies to maintain the size of the test.[33]

A secondary analysis will adjust for pre-specified baseline factors associated with poor psychological outcome (e.g. sedation) and ability to resource and deliver the intervention (e.g. size of critical care unit, teaching status) at both patient and site level. Results of the GLMMs will be reported as differences in means, 95% confidence intervals and p-values.

For peer review only - http://bmjopen.bmj.com/site/about/guidelines.xhtml

Analyses of secondary outcomes will be conducted using GLMMs, with the identity link (i.e. linear regression) for continuous secondary outcomes, reported as differences in means, and the logit link (i.e. logistic regression) for binary secondary outcomes, reported as odds ratios.

The above analyses will evaluate the effectiveness of the intervention among all patients meeting the inclusion criteria and consenting to follow-up, based on the intention to treat principle. A further secondary analysis will use structural mean models with an instrumental variable of allocated treatment to estimate the efficacy (adherence adjusted causal effect) of the stress support sessions among those patients consenting to psychological assessment and stress support sessions, assessed as being at high risk of psychological morbidity and receiving stress support sessions.[34]

Economic evaluation

A cost-effectiveness analysis (CEA) will be undertaken to assess the relative cost-effectiveness of the intervention versus usual care. Resource use and outcome data will be used to report cost-effectiveness at six months and to project the lifetime cost-effectiveness of each strategy.

The cost analysis will take a health and personal health services perspective. Resource use data will be combined with unit costs from the NHS Payment by Results database and from local Trust Finance Departments, to report the total costs per patient at six months for intervention versus usual care.[35 36]

HRQoL data from the EQ-5D-5L questionnaires at six months will be combined with survival data using linear interpolation to report QALYs at six months. The CEA will follow the intention-to-treat principle and report the mean (95% confidence interval) incremental costs, QALYs and net monetary benefit at six months.

The CEA will use multilevel linear regression models that allow for clustering[37] including a random effect of site and a fixed effect of period. The analysis will adjust for pre-specified baseline covariates at both patient and site level.

Lifetime cost-effectiveness will be projected by encapsulating the relative effects of the alternative strategies on long-term survival and HRQoL, combining extrapolations from the patient survival data, with external evidence on long-term survival and HRQoL.[38 39] The long-term survival of patients will be extrapolated from the cluster-RCT data by fitting alternative parametric survival curves (e.g. Weibull, exponential, lognormal, log logistic and Gompertz) to the observed survival data. The method of parametric extrapolation of survival for the base case will be chosen on the basis of model fit and plausibility when compared with age-gender matched general population survival.[40] Survival will then be extrapolated according to chosen parametric function for the duration of years that parametric curves predicts excess mortality compared to age-gender matched general population, after which we will assume that all cause death rates were those of the age-gender matched general population. In the base case, quality of life calculated at six months will be assumed to apply to each subsequent year of life, after allowing for decrements in quality of life according to advancing age. We will project lifetime costs by applying morbidity costs estimated at six months over the period of excess mortality. Predicted survival and HRQoL will be combined to report lifetime QALYs, and to project lifetime incremental costs, incremental QALYs, and incremental net benefits for the alternative strategies of

For peer review only - http://bmjopen.bmj.com/site/about/guidelines.xhtml

For peer review only

care. Sensitivity analyses will test whether the results are robust to methodological assumptions (e.g. specification of the statistical model, extrapolation approach, alternative HRQoL assumptions, and learning curve effects).

Process evaluation

The process evaluation data will be analysed using a combination of qualitative and quantitative methods to measure and understand the reason for any variation in the delivery of the intervention across sites. Interview data will be transcribed and analysed using a 7-stepped Framework approach[41] which includes coding the data, developing and applying an analytical framework, and producing data summaries. A sample of transcripts will be double-coded and reviewed by another independent member of the research team to ensure trustworthiness and confirmability. Data summaries will be interpreted and used to construct overall explanations of the data by two members of the research team.

Analysis of the process evaluation data will be conducted before the outcome evaluation to avoid any bias in the interpretation of the data, and to generate hypotheses that may be subsequently tested in statistical analyses of integrated process and outcome data. The process evaluation data will be combined with the trial outcome data to uncover the relationship between the variation in intervention delivery and trial outcomes.

POWER CALCULATION

Pre-trial power calculation

The power calculation was completed using the approach of Hussey & Hughes (2007)[33] to achieve 90% power to detect a reduction from 6 points to 3.1 points ($p<0.05$) in the mean PSS-SR at six months, and was based on the following assumptions:

- Mean (6) and standard deviation (7.5) of the PSS-SR were taken from patients in the feasibility study.
- Estimated intra-cluster correlation (ICC) of 0.138 – between-site coefficient of variation 0.5 corresponding to between-site standard deviation of 3 (conservative estimate as no multi-centre data available).[42] Note: the inclusion of a baseline recruitment period means that the sample size calculation is less sensitive to the degree of clustering.[33]
- Treatment effect of a reduction of 2.9 points on the PSS-SR based on: reliable change index for the PSS-SR of 8.6 points[43] being observed in 40% of eligible patients in the intervention periods assessed as being at high risk of psychological morbidity using the IPAT, with 16% of recruiting patients declining the intervention.
- Harmonic mean of the number of patients completing follow-up (52 per site per annum – corresponding to 22 in a five-month period) based on data from the CMP.

With the design and the above assumptions, the estimated total number of patients recruited (based on CMP data) for the RCT would be 1,914 patients from the twenty-four sites. It is anticipated that 438 will be assessed using the IPAT, of which 175 (40%) will be assessed as being at high risk of psychological morbidity and receive the stress support sessions.

For peer review only - http://bmjopen.bmj.com/site/about/guidelines.xhtml

Final review of assumptions in pre-trial power calculation

During recruitment, in consultation with the Trial Steering Committee (TSC) and Data Monitoring and Ethics Committee (DMEC), a review of assumptions underlying the pre-trial power calculation was conducted once outcome data were available for patients recruited during the five-month baseline period in both intervention group and control group sites. This review, undertaken using data available on 9 August 2016, identified the following re-estimations of the assumptions:

- Mean (10.3) and standard deviation (10.8) of the PSS-SR.
- ICC of 0.087 (95% confidence interval 0 to 0.192) [with mean, standard deviation and ICC estimated using all available data from a previous observational study, the feasibility study and the baseline period of the cluster-RCT].
- Treatment effect of a reduction of 4.2 points on the PSS-SR – estimated by retaining the same effect size as a multiple of the within-site standard deviation.
- Harmonic mean of the number of patients completing follow-up (30.7 per site per annum – corresponding to 12.8 in a five-month period) estimated using observed data from the baseline period.

This review established that the planned design had an anticipated 78% power under the observed parameter estimates (allowing for uncertainty in the between-site variation, between 73% and 85% power).

Consequently, the decision was taken to extend recruitment in all sites to the end of planned recruitment for the final group of eight sites (corresponding to an harmonic mean of 16.5 patients completing follow-up per site during the intervention period, allowing for the variation from five months to nine months duration across sites, Figure 1). With this extension to recruitment, the planned design had an anticipated 85% power (allowing for uncertainty in the between-site variation, between 79% and 91% power). It was anticipated that, with this extension to recruitment, the estimated total number of patients recruited would be 1,378. Recruitment continued to be monitored to ensure 1,378 or more patients were recruited. A final decision to extend recruitment by an additional two months in all sites was taken to ensure this minimum number was achieved. A protocol amendment was implemented to reflect this review of assumptions and the extension to the recruitment period.

ETHICS AND OVERSIGHT

Ethics

The Protocol and other trial-related documentation (and any amendments) received favourable ethical opinion from NRES Committee South Central - Oxford B Research Ethics Committee (reference: 15/SC/0287). The full Protocol (including amendments) is available on the NIHR website.[44] All participating sites obtained local approvals prior to patient recruitment.

Oversight

For peer review only - http://bmjopen.bmj.com/site/about/guidelines.xhtml

The Trial Management Group is responsible for the management of POPPI and is led by Professor Kathryn Rowan (Chief Investigator) and Paul Mouncey (Senior Researcher). In addition, the TMG comprises the trial investigators and relevant ICNARC CTU staff, who meet regularly to discuss the progress of the trial. ICNARC is trial sponsor (contact details available on www.icnarc.org), and the ICNARC CTU is responsible for day-to-day trial management, is the data custodian, and will conduct central and on-site monitoring of sites and data. POPPI is managed according to the principles of the International Conference on Harmonisation (ICH) Good Clinical Practice (GCP). The ICNARC CTU will act to preserve patient confidentiality and will not disclose or reproduce any information by which patients could be identified. Any patient identifiable data leaving sites will be encrypted to ensure anonymity. All procedures for handling, processing, storing and destroying data are compliant with the Data Protection Act 1998.

A TSC and a DMEC have been convened as trial oversight committees. The TSC is independently chaired by Professor Sallie Lamb and includes critical care clinicians, psychologists, patient and public involvement representatives, along with the Chief Investigator and Lead Clinical Investigator. The TSC monitors trial progress and at least 75% of members are independent. The independent DMEC is chaired by Professor Marion Campbell and also includes an experienced critical care clinician and an experienced statistician. The DMEC is independent of both the trial team, sponsor and TSC, and operates under the DAMOCLES Charter[45], reporting to the TSC, making recommendations on the continuation, or not, of the trial.

TRIAL STATUS

This paper presents the protocol (version 2.2, 6 March 2017) for the first, large, multi-centre cluster-RCT evaluating a complex intervention commencing in the critical care unit and aimed at reducing longer-term psychological morbidity for critical care survivors in the UK. The first participant was recruited in September 2015. At the time of first manuscript submission, data collection for the trial was ongoing and due to be complete in December 2017. The trial results will be disseminated in 2018 through presentations at national and international conferences and publication in peer reviewed medical journals.

For peer review only

For peer review only - http://bmjopen.bmj.com/site/about/guidelines.xhtml

ACKNOWLEDGEMENTS

We thank the following people for their contributions to the set-up and delivery of the POPPI trial: Nick Hudson, Helen Antwi, Steven Saunders, Robert Bradley, Sian Martin, Imran Khan, Jerome Wulff, Bronagh Blackwood, Donatella D'Antoni, Nicole Als, Margaret Harvey, Chris Whitman and David Aaronovitch. Research and clinical staff at the 24 participating sites: Bristol Royal Infirmary, Countess of Chester Hospital, Darlington Memorial Hospital, Freeman Hospital, Hull Royal Infirmary, James Cook University Hospital, Medway Maritime Hospital, Musgrove Park Hospital, Peterborough City Hospital, Poole Hospital, Queen Alexandra Hospital, Queen Elizabeth Hospital (King's Lynn), Queen's Medical Centre, Royal Berkshire Hospital, Royal Cornwall Hospital, Royal Gwent Hospital, St George's Hospital, St James's University Hospital, The Ipswich Hospital, Ulster Hospital, University Hospital Coventry, Warwick Hospital, Whiston Hospital and York Hospital.

AUTHORS' CONTRIBUTIONS

KR, DW, JWei, CB, DS, JWel, DHo, MM, DHa, SH, RG, and ZS conceived the POPPI trial. KR led the design, grant application and, as Chief Investigator, had oversight for the trial. DW, as Lead Clinical Investigator, led development and delivery of the intervention with input from DS, JWel and ARB; PM, as Trial Manager, managed the trial with support from ARB; DHa designed and has oversight for the statistical analysis; RG and ZS designed and have oversight for the economic analysis; LE designed and conducted the process evaluation. ARB drafted this manuscript and all authors read and approved the final version.

FUNDING AND SPONSORSHIP

This project was funded by the National Institute for Health Research (NIHR) Health Services and Delivery Research (HS&DR) Programme (project number: 12/64/124). The Intensive Care National Audit & Research Centre (ICNARC) are the sponsor for the trial. The views and opinions expressed therein are those of the authors and do not necessarily reflect those of the HS&DR Programme, NIHR, NHS the Department of Health nor the sponsor.

COMPETING INTERESTS

None declared.

For peer review only - http://bmjopen.bmj.com/site/about/guidelines.xhtml

FIGURES LEGENDS

Figure 1. POPPI Cluster–Randomised Clinical Trial schedule

Figure 2. Site timeline during transition period

Figure 3. Patient timeline during intervention period

For peer review only

For peer review only - http://bmjopen.bmj.com/site/about/guidelines.xhtml

TABLES

Table 1. Patient data collection schedule

	**Baseline (at point of recruitment)**	**End of critical care unit stay**	Intervention group sites – during transition and intervention period			**Six months post-recruitment**
			**Before SSS-I**	**During sessions**	**After SSS-III**	
Collected in-hospital						
**Patient details**	✓					
**Clinical/baseline data**	✓					
**Critical care unit stay**		✓				
**IPAT assessment**			✓			
**SSS delivery**				✓		
**STAI**	✓				✓	
Collected via follow-up questionnaires sent to patients						
**PSS-SR**						✓
**HADS**						✓
**EQ-5D-5L**						✓
**Health service and resource use**						✓

SSS denotes stress support session(s). IPAT denotes Intensive care Psychological Assessment Tool. STAI denotes State-Trait Anxiety Inventory. PSS-SR denotes PTSD Symptom Scale - Self Report version. HADS denotes Hospital Anxiety and Depression Scale. EQ-5D-5L denotes EuroQoL health questionnaire.

For peer review only

REFERENCES

1. ICNARC. Key statistics from the Case Mix Programme — adult, general critical care units 2017 [Available from: https://www.icnarc.org/DataServices/Attachments/Download/a30185e2-0e19-e711-80e6-1402ec3fcd79 accessed 23/11/2017.
2. Wade DM, Howell DC, Weinman JA, et al. Investigating risk factors for psychological morbidity three months after intensive care: a prospective cohort study. *Crit Care* 2012;16(5):R192. doi: 10.1186/cc11677 [published Online First: 2012/10/17]
3. Ely EW, Siegel MD, Inouye SK. Delirium in the intensive care unit: an under-recognized syndrome of organ dysfunction. *Semin Respir Crit Care Med* 2001;22(2):115-26. doi: 10.1055/s-2001-13826 [published Online First: 2005/08/10]
4. Wade D, Hardy R, Howell D, et al. Identifying clinical and acute psychological risk factors for PTSD after critical care: a systematic review. *Minerva Anestesiol* 2013;79(8):944-63. [published Online First: 2013/04/06]
5. Jones C, Backman C, Capuzzo M, et al. Precipitants of post-traumatic stress disorder following intensive care: a hypothesis generating study of diversity in care. *Intensive Care Med* 2007;33(6):978-85. doi: 10.1007/s00134-007-0600-8 [published Online First: 2007/03/27]
6. Novaes MA, Knobel E, Bork AM, et al. Stressors in ICU: perception of the patient, relatives and health care team. *Intensive Care Med* 1999;25(12):1421-6. [published Online First: 2000/02/08]
7. Jones C, Griffiths RD, Humphris G, et al. Memory, delusions, and the development of acute posttraumatic stress disorder-related symptoms after intensive care. *Crit Care Med* 2001;29(3):573-80. [published Online First: 2001/05/25]
8. Wade DM, Brewin CR, Howell DC, et al. Intrusive memories of hallucinations and delusions in traumatized intensive care patients: An interview study. *Br J Health Psychol* 2014 doi: 10.1111/bjhp.12109 [published Online First: 2014/06/20]
9. Samuelson KA, Lundberg D, Fridlund B. Stressful memories and psychological distress in adult mechanically ventilated intensive care patients - a 2-month follow-up study. *Acta Anaesthesiol Scand* 2007;51(6):671-8. doi: 10.1111/j.1399-6576.2007.01292.x [published Online First: 2007/06/15]
10. Granja C, Gomes E, Amaro A, et al. Understanding posttraumatic stress disorder-related symptoms after critical care: the early illness amnesia hypothesis. *Crit Care Med* 2008;36(10):2801-9. doi: 10.1097/CCM.0b013e318186a3e7 [published Online First: 2008/09/04]
11. Myhren H, Toien K, Ekeberg O, et al. Patients' memory and psychological distress after ICU stay compared with expectations of the relatives. *Intensive Care Med* 2009;35(12):2078-86. doi: 10.1007/s00134-009-1614-1 [published Online First: 2009/09/17]
12. Davydow DS, Zatzick D, Hough CL, et al. A longitudinal investigation of posttraumatic stress and depressive symptoms over the course of the year following medical-surgical intensive care unit admission. *Gen Hosp Psychiatry* 2013;35(3):226-32. doi: 10.1016/j.genhosppsych.2012.12.005 [published Online First: 2013/02/02]
13. Davydow DS, Zatzick D, Hough CL, et al. In-hospital acute stress symptoms are associated with impairment in cognition 1 year after intensive care unit admission. *Ann Am Thorac Soc* 2013;10(5):450-7. doi: 10.1513/AnnalsATS.201303-060OC [published Online First: 2013/08/31]
14. Ratzer M, Romano E, Elklit A. Posttraumatic Stress Disorder in Patients Following Intensive Care Unit Treatment: A Review of Studies Regarding Prevalence and Risk Factors. *Trauma & Treatment* 2014;190(3) doi: 10.4172/2167-1222.1000190
15. Davydow DS, Gifford JM, Desai SV, et al. Depression in general intensive care unit survivors: a systematic review. *Intensive Care Med* 2009;35(5):796-809. doi: 10.1007/s00134-009-1396-5 [published Online First: 2009/01/24]
16. Bienvenu OJ, Colantuoni E, Mendez-Tellez PA, et al. Depressive symptoms and impaired physical function after acute lung injury: a 2-year longitudinal study. *Am J Respir Crit Care Med* 2012;185(5):517-24. doi: 10.1164/rccm.201103-0503OC [published Online First: 2011/12/14]
17. Ballenger JC, Davidson JR, Lecrubier Y, et al. Consensus statement on posttraumatic stress disorder from the International Consensus Group on Depression and Anxiety. *J Clin Psychiatry* 2000;61 Suppl 5:60-6. [published Online First: 2000/04/13]
18. Boscarino JA. A prospective study of PTSD and early-age heart disease mortality among Vietnam veterans: implications for surveillance and prevention. *Psychosom Med* 2008;70(6):668-76. doi: 10.1097/PSY.0b013e31817bccaf [published Online First: 2008/07/04]
19. Dedert EA, Calhoun PS, Watkins LL, et al. Posttraumatic stress disorder, cardiovascular, and metabolic disease: a review of the evidence. *Ann Behav Med* 2010;39(1):61-78. doi: 10.1007/s12160-010-9165-9 [published Online First: 2010/02/23]
20. Azoulay E, Pochard F, Kentish-Barnes N, et al. Risk of post-traumatic stress symptoms in family members of intensive care unit patients. *Am J Respir Crit Care Med* 2005;171(9):987-94. doi: 10.1164/rccm.200409-1295OC [published Online First: 2005/01/25]
21. Cuthbertson BH, Rattray J, Campbell MK, et al. The PRaCTICaL study of nurse led, intensive care follow-up programmes for improving long term outcomes from critical illness: a pragmatic randomised controlled trial. *BMJ* 2009;339:b3723. doi: 10.1136/bmj.b3723 [published Online First: 2009/10/20]

For peer review only - http://bmjopen.bmj.com/site/about/guidelines.xhtml

22. Hatch R, McKechnie S, Griffiths J. Psychological intervention to prevent ICU-related PTSD: who, when and for how long? *Crit Care* 2011;15(2):141. doi: 10.1186/cc10054 [published Online First: 2011/05/06]
23. Wade DM, Hankins M, Smyth DA, et al. Detecting acute distress and risk of future psychological morbidity in critically ill patients: validation of the intensive care psychological assessment tool. *Crit Care* 2014;18(5):519. doi: 10.1186/s13054-014-0519-8 [published Online First: 2014/09/25]
24. Jones C, Skirrow P, Griffiths RD, et al. Rehabilitation after critical illness: a randomized, controlled trial. *Crit Care Med* 2003;31(10):2456-61. doi: 10.1097/01.ccm.0000089938.56725.33 [published Online First: 2003/10/08]
25. Peris A, Bonizzoli M, Iozzelli D, et al. Early intra-intensive care unit psychological intervention promotes recovery from post traumatic stress disorders, anxiety and depression symptoms in critically ill patients. *Crit Care* 2011;15(1):R41. doi: 10.1186/cc10003 [published Online First: 2011/01/29]
26. Sessler CN, Gosnell MS, Grap MJ, et al. The Richmond Agitation-Sedation Scale: validity and reliability in adult intensive care unit patients. *Am J Respir Crit Care Med* 2002;166(10):1338-44. doi: 10.1164/rccm.2107138 [published Online First: 2002/11/08]
27. Spielberger CD, Gorsuch RL, Lushene R, et al. Manual for the State-Trait Anxiety Inventory. Palo Alto, CA: Consulting Psychologists Press 1983.
28. Foa EB, Cashman L, Jaycox L, et al. The validation of a self-report measure of posttraumatic stress disorder: the Posttraumatic Diagnostic Scale. *Psychol Assess* 1997;9:445-51.
29. Zigmond AS, Snaith RP. The hospital anxiety and depression scale. *Acta Psychiatr Scand* 1983;67(6):361-70. [published Online First: 1983/06/01]
30. Herdman M, Gudex C, Lloyd A, et al. Development and preliminary testing of the new five-level version of EQ-5D (EQ-5D-5L). *Qual Life Res* 2011;20(10):1727-36. doi: 10.1007/s11136-011-9903-x [published Online First: 2011/04/12]
31. Ehring T, Kleim B, Clark DM, et al. Screening for posttraumatic stress disorder: what combination of symptoms predicts best? *J Nerv Ment Dis* 2007;195(12):1004-12. doi: 10.1097/NMD.0b013e31815c1999 [published Online First: 2007/12/20]
32. Ely EW, Inouye SK, Bernard GR, et al. Delirium in mechanically ventilated patients: validity and reliability of the confusion assessment method for the intensive care unit (CAM-ICU). *JAMA* 2001;286(21):2703-10. [published Online First: 2001/12/26]
33. Hussey MA, Hughes JP. Design and analysis of stepped wedge cluster randomized trials. *Contemp Clin Trials* 2007;28(2):182-91. doi: 10.1016/j.cct.2006.05.007 [published Online First: 2006/07/11]
34. Maracy M, Dunn G. Estimating dose-response effects in psychological treatment trials: the role of instrumental variables. *Stat Methods Med Res* 2011;20(3):191-215. doi: 10.1177/0962280208097243 [published Online First: 2008/11/28]
35. Curtis L. Unit costs of health & social care 2011. : . Canterbury, UK: Personal Service Research Unit, University of Kent 2011.
36. Department of Health. NHS reference costs 2010-11. London: Department of Health, 2011.
37. Grieve R, Nixon R, Thompson SG. Bayesian hierarchical models for cost-effectiveness analyses that use data from cluster randomized trials. *Med Decis Making* 2010;30(2):163-75. doi: 10.1177/0272989x09341752 [published Online First: 2009/08/14]
38. Wright JC, Plenderleith L, Ridley SA. Long-term survival following intensive care: subgroup analysis and comparison with the general population. *Anaesthesia* 2003;58(7):637-42. [published Online First: 2003/06/07]
39. Green C, Dinnes J, Takeda AL, et al. Evaluation of the cost-effectiveness of drotrecogin alfa (activated) for the treatment of severe sepsis in the United Kingdom. *Int J Technol Assess Health Care* 2006;22(1):90-100. [published Online First: 2006/05/06]
40. Latimer NR. Survival Analysis for Economic Evaluations Alongside Clinical Trials—Extrapolation with Patient-Level Data: Inconsistencies, Limitations, and a Practical Guide. *Med Decis Making* 2013 doi: 10.1177/0272989x12472398
41. Gale NK, Heath G, Cameron E, et al. Using the framework method for the analysis of qualitative data in multi-disciplinary health research. *BMC Med Res Methodol* 2013;13:117. doi: 10.1186/1471-2288-13-117 [published Online First: 2013/09/21]
42. Hayes RJ, Bennett S. Simple sample size calculation for cluster-randomized trials. *Int J Epidemiol* 1999;28(2):319-26. [published Online First: 1999/05/26]
43. Brewin CR, Fuchkan N, Huntley Z, et al. Outreach and screening following the 2005 London bombings: usage and outcomes. *Psychol Med* 2010;40(12):2049-57. doi: 10.1017/s0033291710000206 [published Online First: 2010/02/25]
44. National Institute for Health Research. Psychological Outcomes following a nurse-led Preventative Psychological Intervention for critically ill patients (POPPI) Study [Available from: https://www.journalslibrary.nihr.ac.uk/programmes/hsdr/1264124/#/ accessed 04/12/2017.
45. A proposed charter for clinical trial data monitoring committees: helping them to do their job well. *Lancet* 2005;365(9460):711-22. doi: 10.1016/s0140-6736(05)17965-3 [published Online First: 2005/02/22]

For peer review only - http://bmjopen.bmj.com/site/about/guidelines.xhtml

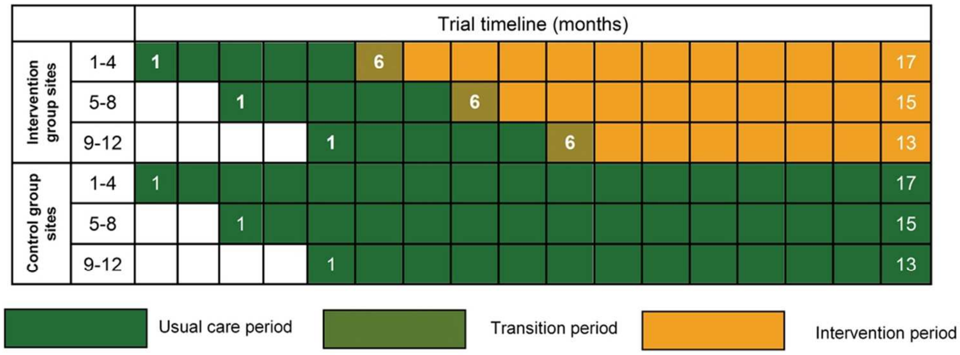

94x37mm (300 x 300 DPI)

For peer review only

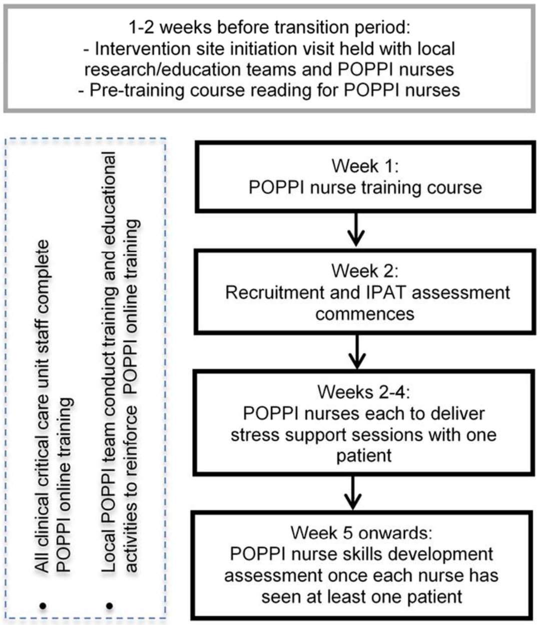

114x76mm (300 x 300 DPI)

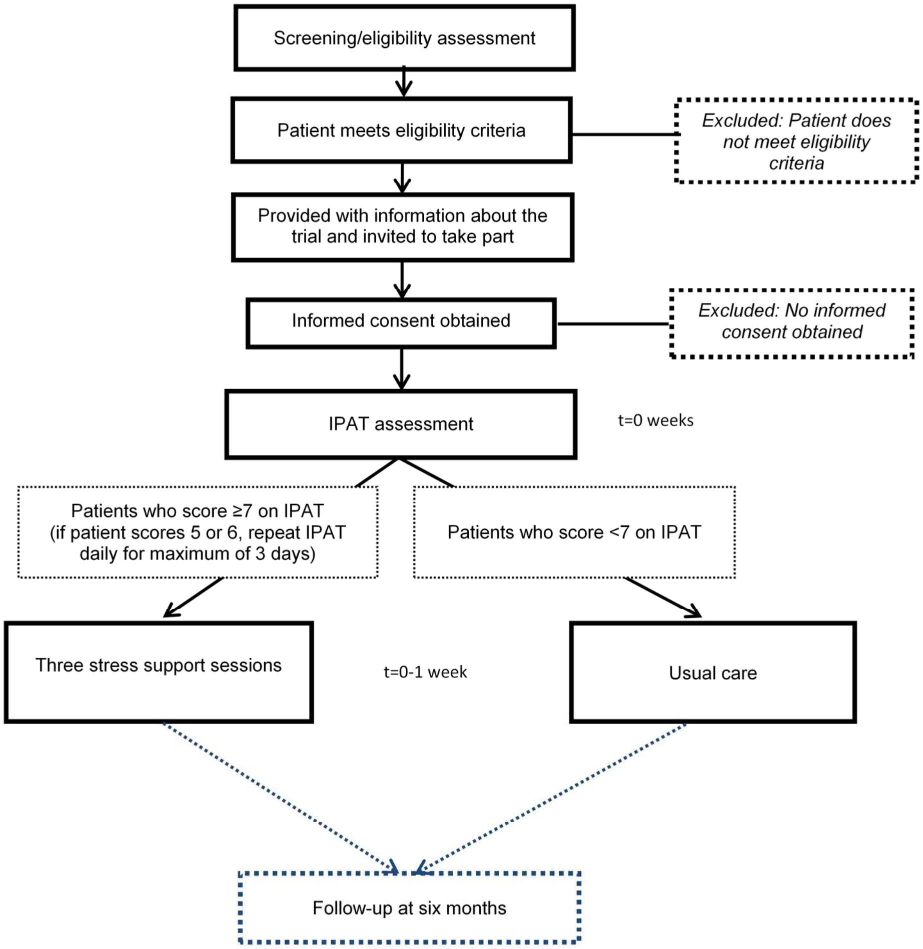

IPAT denotes Intensive care Psychological Assessment Tool. STAI denotes State-Trait Anxiety Inventory. SSS denotes stress support session.

183x194mm (300 x 300 DPI)

SPIRIT

STANDARD PROTOCOL ITEMS: RECOMMENDATIONS FOR INTERVENTIONAL TRIALS

SPIRIT 2013 Checklist: Recommended items to address in a clinical trial protocol and related documents*

Section/item	Item No	Description	Addressed on page number
**Administrative information**			
Title	1	Descriptive title identifying the study design, population, interventions, and, if applicable, trial acronym	1
Trial registration	2a	Trial identifier and registry name. If not yet registered, name of intended registry	3
	2b	All items from the World Health Organization Trial Registration Data Set	Throughout
Protocol version	3	Date and version identifier	15
Funding	4	Sources and types of financial, material, and other support	16
Roles and responsibilities	5a	Names, affiliations, and roles of protocol contributors	1, 16
	5b	Name and contact information for the trial sponsor	15
	5c	Role of study sponsor and funders, if any, in study design; collection, management, analysis, and interpretation of data; writing of the report; and the decision to submit the report for publication, including whether they will have ultimate authority over any of these activities	15, 16
	5d	Composition, roles, and responsibilities of the coordinating centre, steering committee, endpoint adjudication committee, data management team, and other individuals or groups overseeing the trial, if applicable (see Item 21a for data monitoring committee)	14, 15

For peer review only

For peer review only - http://bmjopen.bmj.com/site/about/guidelines.xhtml

**Introduction**			
Background and rationale	6a	Description of research question and justification for undertaking the trial, including summary of relevant studies (published and unpublished) examining benefits and harms for each intervention	4
	6b	Explanation for choice of comparators	6
Objectives	7	Specific objectives or hypotheses	5
Trial design	8	Description of trial design including type of trial (eg, parallel group, crossover, factorial, single group), allocation ratio, and framework (eg, superiority, equivalence, noninferiority, exploratory)	5, 11
**Methods: Participants, interventions, and outcomes**			
Study setting	9	Description of study settings (eg, community clinic, academic hospital) and list of countries where data will be collected. Reference to where list of study sites can be obtained	16
Eligibility criteria	10	Inclusion and exclusion criteria for participants. If applicable, eligibility criteria for study centres and individuals who will perform the interventions (eg, surgeons, psychotherapists)	5, 6, 8
Interventions	11a	Interventions for each group with sufficient detail to allow replication, including how and when they will be administered	8, 9
	11b	Criteria for discontinuing or modifying allocated interventions for a given trial participant (eg, drug dose change in response to harms, participant request, or improving/worsening disease)	9
	11c	Strategies to improve adherence to intervention protocols, and any procedures for monitoring adherence (eg, drug tablet return, laboratory tests)	9
	11d	Relevant concomitant care and interventions that are permitted or prohibited during the trial	n/a
Outcomes	12	Primary, secondary, and other outcomes, including the specific measurement variable (eg, systolic blood pressure), analysis metric (eg, change from baseline, final value, time to event), method of aggregation (eg, median, proportion), and time point for each outcome. Explanation of the clinical relevance of chosen efficacy and harm outcomes is strongly recommended	10
Participant timeline	13	Time schedule of enrolment, interventions (including any run-ins and washouts), assessments, and visits for participants. A schematic diagram is highly recommended (see Figure)	6, 8, 9, 10, Figure 1, Figure 3

For peer review only - http://bmjopen.bmj.com/site/about/guidelines.xhtml

Sample size	14	Estimated number of participants needed to achieve study objectives and how it was determined, including clinical and statistical assumptions supporting any sample size calculations	13, 14
Recruitment	15	Strategies for achieving adequate participant enrolment to reach target sample size	8
**Methods: Assignment of interventions (for controlled trials)**			
Allocation:			
Sequence generation	16a	Method of generating the allocation sequence (eg, computer-generated random numbers), and list of any factors for stratification. To reduce predictability of a random sequence, details of any planned restriction (eg, blocking) should be provided in a separate document that is unavailable to those who enrol participants or assign interventions	6
Allocation concealment mechanism	16b	Mechanism of implementing the allocation sequence (eg, central telephone; sequentially numbered, opaque, sealed envelopes), describing any steps to conceal the sequence until interventions are assigned	6
Implementation	16c	Who will generate the allocation sequence, who will enrol participants, and who will assign participants to interventions	6
Blinding (masking)	17a	Who will be blinded after assignment to interventions (eg, trial participants, care providers, outcome assessors, data analysts), and how	n/a
	17b	If blinded, circumstances under which unblinding is permissible, and procedure for revealing a participant's allocated intervention during the trial	n/a
**Methods: Data collection, management, and analysis**			
Data collection methods	18a	Plans for assessment and collection of outcome, baseline, and other trial data, including any related processes to promote data quality (eg, duplicate measurements, training of assessors) and a description of study instruments (eg, questionnaires, laboratory tests) along with their reliability and validity, if known. Reference to where data collection forms can be found, if not in the protocol	10, 11
	18b	Plans to promote participant retention and complete follow-up, including list of any outcome data to be collected for participants who discontinue or deviate from intervention protocols	9, 10

For peer review only - http://bmjopen.bmj.com/site/about/guidelines.xhtml

Data management	19	Plans for data entry, coding, security, and storage, including any related processes to promote data quality (eg, double data entry; range checks for data values). Reference to where details of data management procedures can be found, if not in the protocol	11
Statistical methods	20a	Statistical methods for analysing primary and secondary outcomes. Reference to where other details of the statistical analysis plan can be found, if not in the protocol	11, 12, 13
	20b	Methods for any additional analyses (eg, subgroup and adjusted analyses)	11, 12, 13
	20c	Definition of analysis population relating to protocol non-adherence (eg, as randomised analysis), and any statistical methods to handle missing data (eg, multiple imputation)	12
**Methods: Monitoring**			
Data monitoring	21a	Composition of data monitoring committee (DMC); summary of its role and reporting structure; statement of whether it is independent from the sponsor and competing interests; and reference to where further details about its charter can be found, if not in the protocol. Alternatively, an explanation of why a DMC is not needed	15
	21b	Description of any interim analyses and stopping guidelines, including who will have access to these interim results and make the final decision to terminate the trial	n/a
Harms	22	Plans for collecting, assessing, reporting, and managing solicited and spontaneously reported adverse events and other unintended effects of trial interventions or trial conduct	n/a
Auditing	23	Frequency and procedures for auditing trial conduct, if any, and whether the process will be independent from investigators and the sponsor	15
**Ethics and dissemination**			
Research ethics approval	24	Plans for seeking research ethics committee/institutional review board (REC/IRB) approval	14
Protocol amendments	25	Plans for communicating important protocol modifications (eg, changes to eligibility criteria, outcomes, analyses) to relevant parties (eg, investigators, REC/IRBs, trial participants, trial registries, journals, regulators)	14

For peer review only - http://bmjopen.bmj.com/site/about/guidelines.xhtml

Consent or assent	26a	Who will obtain informed consent or assent from potential trial participants or authorised surrogates, and how (see Item 32)	8
	26b	Additional consent provisions for collection and use of participant data and biological specimens in ancillary studies, if applicable	n/a
Confidentiality	27	How personal information about potential and enrolled participants will be collected, shared, and maintained in order to protect confidentiality before, during, and after the trial	15
Declaration of interests	28	Financial and other competing interests for principal investigators for the overall trial and each study site	16
Access to data	29	Statement of who will have access to the final trial dataset, and disclosure of contractual agreements that limit such access for investigators	15
Ancillary and post-trial care	30	Provisions, if any, for ancillary and post-trial care, and for compensation to those who suffer harm from trial participation	9
Dissemination policy	31a	Plans for investigators and sponsor to communicate trial results to participants, healthcare professionals, the public, and other relevant groups (eg, via publication, reporting in results databases, or other data sharing arrangements), including any publication restrictions	15
	31b	Authorship eligibility guidelines and any intended use of professional writers	n/a
	31c	Plans, if any, for granting public access to the full protocol, participant-level dataset, and statistical code	14
**Appendices**			
Informed consent materials	32	Model consent form and other related documentation given to participants and authorised surrogates	Not included, but detailed on page 8
Biological specimens	33	Plans for collection, laboratory evaluation, and storage of biological specimens for genetic or molecular analysis in the current trial and for future use in ancillary studies, if applicable	n/a

*It is strongly recommended that this checklist be read in conjunction with the SPIRIT 2013 Explanation & Elaboration for important clarification on the items. Amendments to the protocol should be tracked and dated. The SPIRIT checklist is copyrighted by the SPIRIT Group under the Creative Commons "Attribution-NonCommercial-NoDerivs 3.0 Unported" license.

For peer review only - http://bmjopen.bmj.com/site/about/guidelines.xhtml
