# Supplementary material for: Psychological Outcomes following a nurse-led Preventative Psychological Intervention for critically ill patients (POPPI): protocol for a cluster-randomised clinical trial of a complex intervention
Source: BMJ Open. 2018 Feb 8;8(2):e020908. doi: 10.1136/bmjopen-2017-020908 (PMC5879925; doi:10.1136/bmjopen-2017-020908)
Supplement: Reviewer comments [file bmjopen-2017-020908.reviewer_comments.pdf]

PEER REVIEW HISTORY

BMJ Open publishes all reviews undertaken for accepted manuscripts. Reviewers are asked to complete a checklist review form (**http://bmjopen.bmj.com/site/about/resources/checklist.pdf**) and are provided with free text boxes to elaborate on their assessment. These free text comments are reproduced below.

ARTICLE DETAILS

**TITLE (PROVISIONAL)**	Psychological Outcomes following a nurse-led Preventative Psychological Intervention for critically ill patients (POPPI) – Protocol for a cluster randomised clinical trial of a complex intervention
**AUTHORS**	Richards-Belle, Alvin; Mouncey, Paul; Wade, Dorothy; Brewin, Chris; Emerson, Lydia; Grieve, Richard; Harrison, David; Harvey, Sheila; Howell, David; Mythen, Monty; Sadique, Zia; Smyth, Deborah; Weinman, John; Welch, John; Rowan, Kathryn

This article was not externally reviewed at BMJ Open. Protocols that have been independently assessed prior to submission to BMJ Open are usually fast-tracked to publication on the grounds that further substantial changes will not be possible. This independent assessment will usually be external, independent review for both a substantial grant award from a non-commercial or government funder and ethics approval.
